# Opposing roles for Egalitarian and Staufen in transport, anchoring and localization of *oskar* mRNA in the *Drosophila* oocyte

**DOI:** 10.1371/journal.pgen.1009500

**Published:** 2021-04-02

**Authors:** Sabine Mohr, Andrew Kenny, Simon T. Y. Lam, Miles B. Morgan, Craig A. Smibert, Howard D. Lipshitz, Paul M. Macdonald

**Affiliations:** 1 Department of Molecular Biosciences, The University of Texas at Austin, Austin, Texas, United States of America; 2 Department of Molecular Genetics, University of Toronto, Toronto, Canada; 3 Department of Biochemistry, University of Toronto, Toronto, Canada; New York University, UNITED STATES

## Abstract

Localization of *oskar* mRNA includes two distinct phases: transport from nurse cells to the oocyte, a process typically accompanied by cortical anchoring in the oocyte, followed by posterior localization within the oocyte. Signals within the *oskar* 3’ UTR directing transport are individually weak, a feature previously hypothesized to facilitate exchange between the different localization machineries. We show that alteration of the SL2a stem-loop structure containing the *oskar* transport and anchoring signal (TAS) removes an inhibitory effect such that *in vitro* binding by the RNA transport factor, Egalitarian, is elevated as is *in vivo* transport from the nurse cells into the oocyte. Cortical anchoring within the oocyte is also enhanced, interfering with posterior localization. We also show that mutation of Staufen recognized structures (SRSs), predicted binding sites for Staufen, disrupts posterior localization of *oskar* mRNA just as in *staufen* mutants. Two SRSs in SL2a, one overlapping the Egalitarian binding site, are inferred to mediate Staufen-dependent inhibition of TAS anchoring activity, thereby promoting posterior localization. The other three SRSs in the *oskar* 3’ UTR are also required for posterior localization, including two located distant from any known transport signal. Staufen, thus, plays multiple roles in localization of *oskar* mRNA.

## Author summary

Within cells, many components must be enriched at specific subcellular locations. One mechanism to achieve the nonrandom distribution of a protein is localization of its cognate mRNA. When localization of an mRNA involves more than one step, the mRNA must be released from the machinery directing the first step to allow the machinery for the next step to perform its function. For the *Drosophila oskar* mRNA the first step is transport of the mRNA from its site of synthesis to the oocyte, where the next step is localization of the mRNA to the posterior pole of the oocyte. Prior studies suggested that the multiple signals in the *oskar* mRNA that mediate transport were individually weak by necessity, to facilitate action by the posterior localization machinery. Here, we describe a mutant version of the *oskar* mRNA whose behavior suggests an overly tenacious association with the transport machinery, leading to failure in posterior localization. Biochemical studies show that the mutant signal has enhanced binding to the Egalitarian RNA binding protein, which mediates transport. Genetic studies show that the Staufen RNA binding protein has an opposing role, facilitating dissociation from the transport machinery to enable posterior localization.

## Introduction

Localization of mRNAs serves to target expression of encoded proteins to specific subcellular domains [1,2]. One extensively studied example is the *oskar* (*osk*) mRNA: through localization, the mRNA becomes positioned at the posterior pole of the developing *Drosophila* oocyte, the site where the OSK protein acts to recruit factors that establish the embryonic germ line and pattern the posterior region of the embryo [3]. Appearance of OSK protein outside this domain leads to the lethal reorganization of the embryo, with duplicated ectopic posterior pattern elements replacing anterior structures to form bicaudal embryos [4,5].

Cellular RNAs have two general roles. They can encode proteins, or they can function as noncoding RNAs (ncRNAs). Unusually, *osk* mRNA does both, with a noncoding role required for progression through oogenesis [6]. At the earlier stages of oogenesis when *osk* ncRNA activity is required, the mRNA is efficiently transported from nurse cells (the sites of transcription) through ring canals into the oocyte; if this transport is disrupted, *osk* ncRNA activity is also disrupted [7]. Thus, both the coding and noncoding roles of *osk* mRNA require some form of localization. Here, we refer to the two phases of *osk* mRNA localization as ‘transport’ (directed movement from nurse cells to oocyte) and ‘localization’ (directed movement within the oocyte to the posterior at later stages of oogenesis).

Transport of *osk* mRNA into the oocyte is mediated by multiple regulatory elements in its 3’ UTR. Those embedded within two stem-loop (SL) structures, SL2a and SL2b, are most critical and deletion of either SL greatly reduces transport [8–11]. The SL2a signal has been mapped at high resolution and corresponds to the most highly conserved portion of the stem structure [11]. Neither SL2a nor SL2b has strong transport activity on its own; if either is added to a reporter mRNA, there is very limited transport to the oocyte [7,10,11]. By contrast, another stem-loop signal—the TLS—that mediates transport of the *fs(1)K10* mRNA from nurse cells to the oocyte is highly active in isolation: addition of the 44 nucleotide TLS to foreign mRNAs consistently confers highly efficient transport [12].

Because transport of *osk* mRNA is essential, it might seem odd that the individual *osk* signals are weak, even if they are collectively strong. A possible explanation of this paradox was suggested by features of the TLS-type transport mechanism and the peculiar properties of *osk* mRNA localization. Importantly, the TLS mediates not only transport to the oocyte but also anchoring [12]. Starting in stage 8 of oogenesis, the mRNA becomes anchored at the anterior, cortical regions of the oocyte after transport. Because the mRNA enters the oocyte at its anterior end, anchoring may be ‘cortical-specific’ rather than ‘anterior-specific’, with the anterior distribution being a consequence of where the newly transported mRNA first encounters the anchoring substrate. If the TLS-type mechanism, which acts on multiple mRNAs and in a variety of cell types [10,13], also mediates transport of *osk* mRNA into the oocyte, then *osk* mRNA should also be restrained at or near the anterior by cortical anchoring. But this is a problem, because *osk* mRNA needs to be displaced from the anterior in the course of its localization to the posterior of the oocyte. We have proposed that individual *osk* transport signals are weak by necessity to facilitate release from the transport and anchoring machinery, thereby allowing the posterior localization machinery to efficiently move the mRNA to its final destination [11]. Support for this model came from adding the strong TLS to the *osk* mRNA, either as a simple addition or as a substitution for SL2a or SL2b: the result is inappropriate anchoring in anterior and lateral cortical regions of the oocyte, together with reduced efficiency of posterior localization of the mRNA.

A prediction of the ‘weak by necessity’ model is that *osk* mRNA transport relies on the same components that act on the TLS, thus conferring not only transport but also anchoring. The TLS is recognized and bound by Egalitarian (EGL), acting in concert with BicaudalD (BICD) [14]. Transport of the complex is driven by Dynein along microtubules [14]. EGL has been shown to be associated with *osk* mRNA, although it remains unknown whether this binding is direct [15]. If EGL binds directly to the *osk* transport signals, this binding may be constrained in some way to account for the weak activity of the individual signals. This constraint might be achieved by, for example, inherently low affinity of the *osk* transport signals for EGL or competition between EGL and inhibitory factors.

Here we report that a specific alteration of SL2a removes an inhibitory influence, dramatically strengthening both transport and cortical anchoring. In the context of *osk* mRNA, the uninhibited transport and anchoring signal (TAS) behaves like the TLS, disrupting posterior localization of the mRNA within the oocyte. In the context of a reporter mRNA (*i*.*e*., lacking sequences that direct posterior localization), the uninhibited TAS directs both transport and cortical anchoring. Release from inhibition in the altered SL2a correlates with enhanced EGL binding *in vitro*. The double-stranded RNA binding protein Staufen (STAU) has been implicated in posterior localization of *osk* mRNA [16–18]. The *osk* mRNA has been shown to be bound by STAU and several ‘STAU recognized structures’ (SRSs) have been predicted in the *osk* 3’ UTR [19]. We show here that mutation of SRSs in *osk* reduces association with STAU. Two SRSs lie within SL2a, with one overlapping the EGL binding site, suggesting that STAU may compete with EGL for binding. Consistent with this model, loss of STAU activity enhances transport and cortical anchoring of reporter mRNAs with SL2a. Additional SRSs elsewhere in the *osk* mRNA 3’ UTR are also required for posterior localization, likely acting by a different mechanism.

## Results

### A mutant form of the SL2a transport signal possesses enhanced transport and anchoring activity

**Fig 1:**
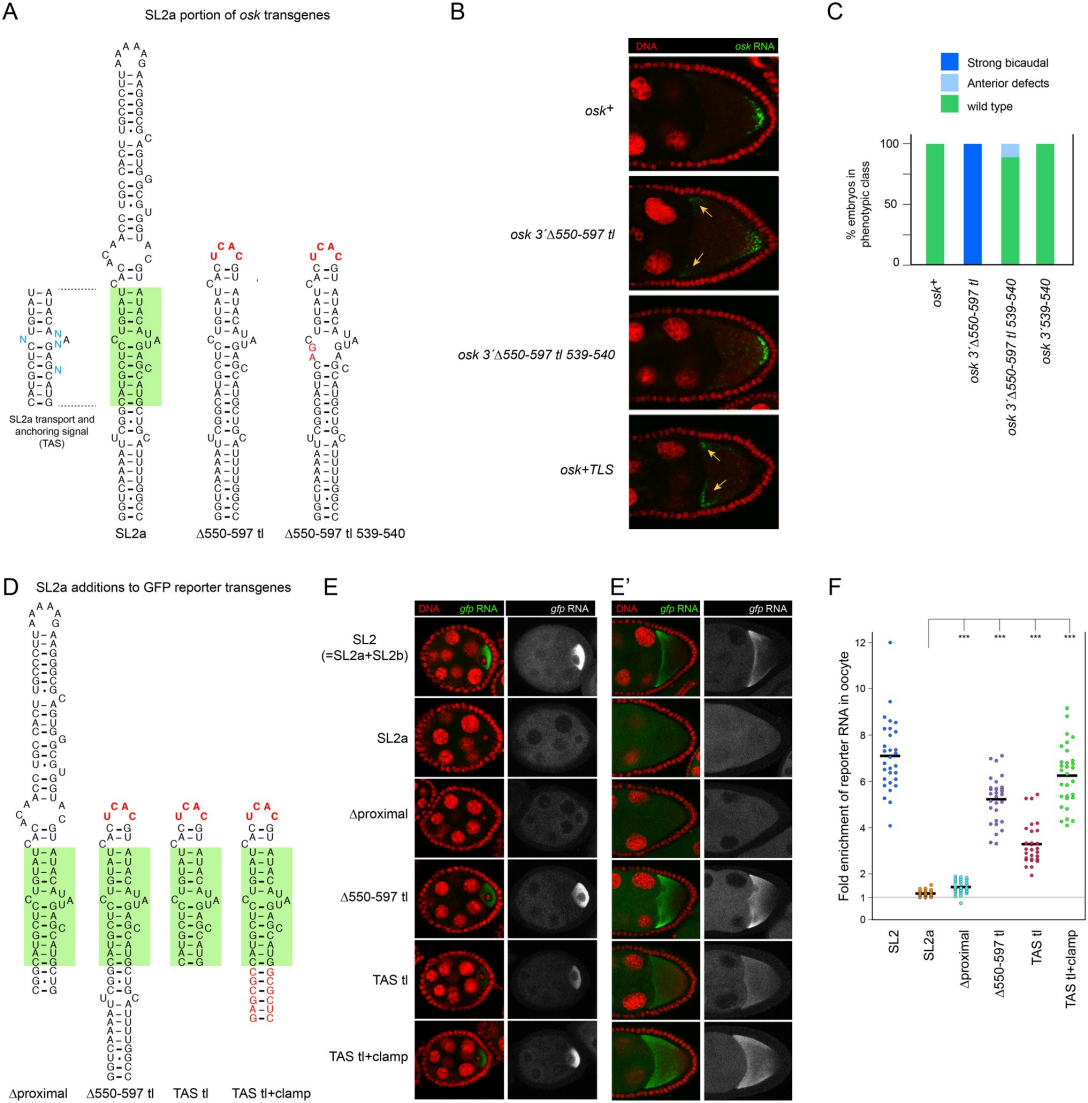
RNA transport and anchoring activity of SL2a and its derivatives. (A) Wild-type and mutant forms of SL2a in genomic *osk* transgenes. Left, the consensus transport and anchoring signal (TAS) ([11]; shaded in green here and in Fig 2), with variable positions (blue) identified by phylogenetic comparisons (S1 Fig). The remainder of the predicted SL2a structure is supported at least in part by mutational analysis and phylogenetic conservation [11]. Mutant structures are shown with altered bases and the terminal tetraloop in red. (B) Distribution of *osk* mRNAs, wild type or mutants from panel A, in early stage 9 egg chambers. Sites of abnormal *osk* mRNA enrichment for this stage are indicated with arrows (orange). All genomic *osk* transgenes, including those in other figures, were present as single copies in *osk* RNA null flies. (C) Axial patterning of embryos from mothers with *osk* genes as in A and B, as well as a control with just the *osk 3’ 539–540* mutations. Mutant phenotypes, from mislocalized *osk* activity, are defined as strong bicaudal (fewer than 3 duplicated abdominal segments) or anterior defects (loss of anterior pattern elements without mirror image duplications). N values for all genotypes were greater than 300. (D) Portions of SL2a included in reporter transgenes expressed under UAS/GAL4 control. Tetraloops and a synthetic stem are in red. (E) Distribution of reporter mRNAs bearing portions of the *osk* mRNA 3’ UTR, including mutant versions from panel D. Left, complete stage 4–6 egg chambers, DNA in red and reporter RNA in green. Right, the green channel from the same images, with identical increase in signal intensity for all genotypes to better reveal reporter mRNA. (E’) Genotypes as in panel E for early stage 9 egg chambers. The right panel is again the green channel alone, but here with no increase in signal intensity relative to the left panel. (F) Quantitation of reporter RNA enrichment in the oocyte for reporter mRNAs tested in panel E. Enrichment was scored as average signal intensity in nurse cells divided by average signal intensity in the oocyte, presented in dot plot format. Absence of oocyte enrichment yields a value of 1, shown as a grey line across the graph. 30 egg chambers were analyzed for each genotype. Statistical significance was evaluated by one-way ANOVA with F(5,174) = 186.2, P < 8 x 10^−68^. Normality was rejected (Bonferroni corrected Shapiro-Wilk test), therefore the Wilcoxon rank sum test was used for *post hoc* analysis. ***: P < 0.01.

In prior work to define the sequence and structure of the SL2a transport signal, we characterized genomic *osk* transgenes with mutant forms of SL2a. Only mutants with alterations within the central, most highly conserved portion of SL2a are defective for transport [11]. Although changes in other portions of SL2a do not substantially impede transport, one mutant, *osk 3*’Δ*550–597 tl* (Fig 1A), was unusual: as oogenesis progressed, the mutant mRNA was inappropriately retained at or near the anterior of the oocyte (Fig 1B). As in some situations when *osk* mRNA is mislocalized to the anterior of the oocyte [4], this ectopic *osk* distribution resulted in formation of bicaudal embryos (Fig 1C).

Because the *osk 3*’Δ*550–597 tl* mRNA distribution was similar to that of *osk* mRNAs to which the strong TLS transport and anchoring signal had been added (Fig 1B) [11], it seemed likely that the abbreviated form of SL2a in *osk 3*’Δ*550–597 tl* possessed enhanced activity, at least for anchoring. If so, introducing a mutation that reduces SL2a activity might be expected to eliminate mRNA mislocalization caused by enhanced anchoring. Mutations in the SL2a transport signal that strongly reduce transport are not suitable for this approach: disruption of *osk* RNA transport to the oocyte inhibits *osk* ncRNA activity and thus causes arrest of oogenesis [7,11], resulting in few to none of the later stage egg chambers in which mislocalization can be assessed. Furthermore, the rare eggs laid fail to develop, eliminating the option of scoring for embryonic patterning defects. However, a more subtle substitution mutation within the SL2a signal (*osk 3’ 539–540*; Fig 1A and 1C) was useful. When incorporated into *osk 3*’Δ*550–597 tl*, the enhanced anchoring phenotypes were dramatically suppressed. At the RNA level, no mislocalized *osk 3*’Δ*550–597 tl 539–540* mRNA could be detected above background (Fig 1B). In a more sensitive biological read out—embryonic patterning—only a small fraction of embryos from *osk 3*’Δ*550–597 tl 539–540* mothers showed evidence of ectopic *osk* activity in the form of weak anterior defects, and no embryos were bicaudal (Fig 1C). Thus, the behavior of the *osk 3*’Δ*550–597 tl* mutant does appear to be due to enhanced activity of the SL2a transport and anchoring signal, referred to henceforth as the TAS.

By analogy to the TLS, the enhanced anchoring activity of the *osk 3*’Δ*550–597 tl* mutant might be expected to be accompanied by increased transport activity. Because of the normally strong transport of *osk* mRNA to the oocyte, a further increase in transport activity would be difficult to detect; therefore, we used a GFP reporter mRNA assay. Addition of wild-type SL2a to a reporter is known to confer very little transport activity, allowing detection of increased transport by SL2a mutants (for comparison, the complete SL2 confers strong transport; Fig 1D–1F) [7,10,11]. As expected, the Δ*550–597 tl* version of SL2a directed robust transport (Fig 1D–1F). In stage 8–9 oocytes the mRNA was enriched in the cortical regions, highest near the anterior, and diminishing towards the posterior (Fig 1E’); thus, it is strongly anchored. We conclude that the TAS behaves much like the TLS and that its anchoring activity is inhibited in the context of the complete SL2a.

Before addressing which changes in the *osk 3*’ Δ*550–597 tl* mutant can relieve inhibition of TAS transport and cortical anchoring activity (below), we first describe additional experiments to ask if other changes in SL2a also affect TAS activity in the GFP reporter assay. In particular, because the TAS lies in the central part of the SL2a stem-loop, we asked if removing the proximal part (in Δproximal; Fig 1D) had the same relief from inhibition as removal of the distal part; however, there was only a slight increase in transport to the oocyte (Fig 1E and 1F). The fraction of Δproximal mutant reporter mRNA in the oocyte, which could only be visualized by enhancing the sensitivity of detection, showed no evidence of cortical anchoring (Fig 1E’). We also tested the isolated TAS (TAS tl), as well as a version with a synthetic extension to the base of the stem, to stabilize folding into a stem-loop (TAS tl+clamp). Both had transport and anchoring activity (Fig 1E and 1F).

### Which changes in the *osk 3*’Δ*550–597 tl* mutant relieve TAS inhibition?

Based on the predicted structure of SL2a (supported by mutational analysis [11]), two general effects, which are not mutually exclusive, may have caused relief of TAS inhibition in the *osk 3*’Δ*550–597 tl* mutant. First, the mutant could be missing a binding site for an inhibitory factor that normally interferes with binding of a transport/anchoring factor such as EGL. The inhibitory factor could, for example, alter TAS folding or bind close to the TAS to inhibit binding by EGL. In the second, the *osk 3*’Δ*550–597 tl* mutant could have enhanced affinity for a transport/anchoring factor independent of any inhibitory factor.

**Fig 2:**
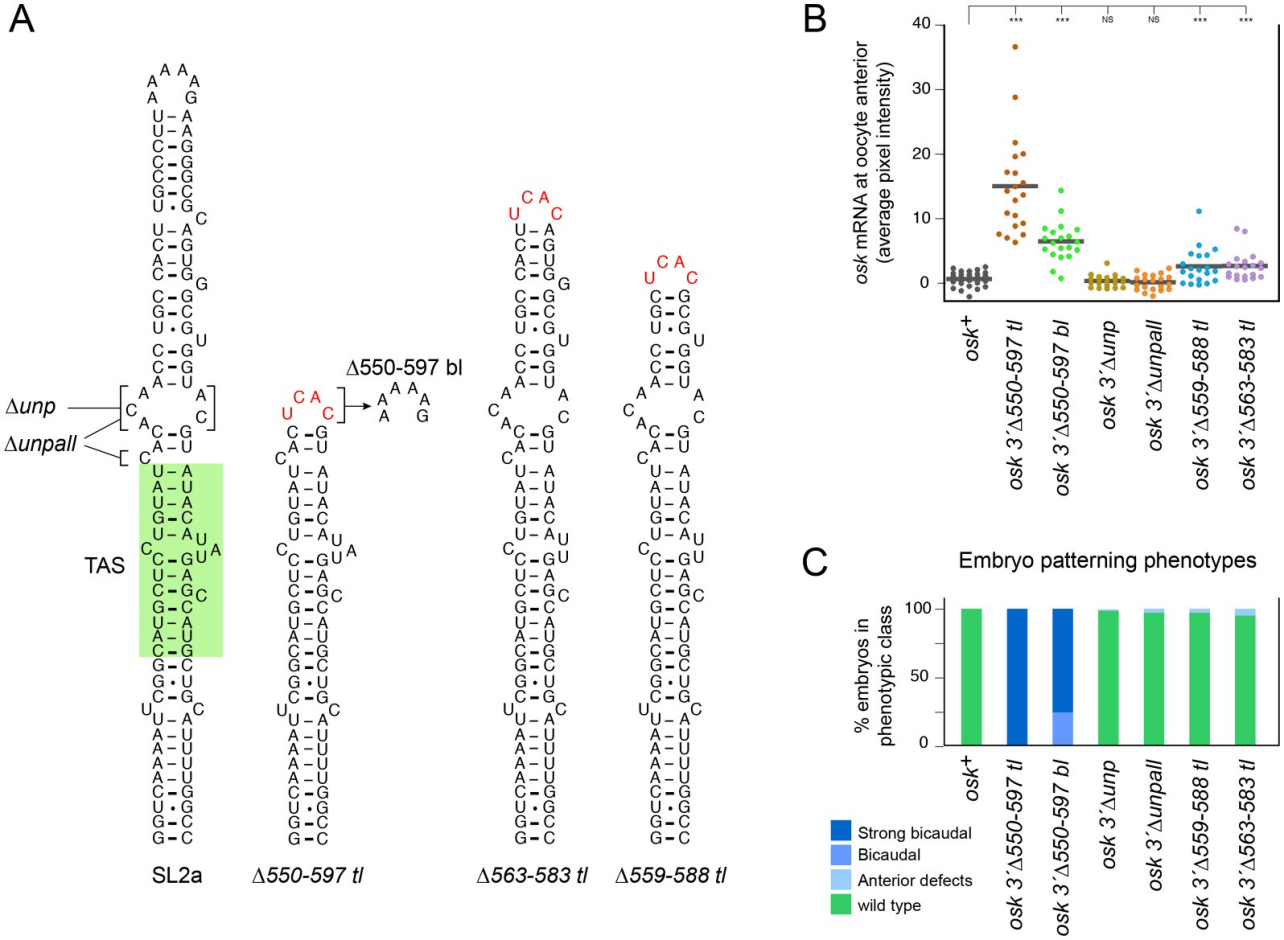
Mutational analysis to identify changes to SL2a that enhance its transport and anchoring activity. (A) Wild-type and mutant forms of SL2a tested in genomic *osk* transgenes as in Fig 1. Changes to wild type, or to the mutant with enhanced transport and anchoring, are indicated on the structures with tetraloops in red. (B) Quantitation of anterior retention of the indicated *osk* transgene mRNAs in late stage 9 oocytes, measured as described in Methods and materials and presented in dot plot format. At least 20 egg chambers were analyzed for each genotype. Statistical significance was evaluated by one-way ANOVA with F(6,143) = 51.90, P < 2 x 10^−33^. Normality was rejected (Bonferroni corrected Shapiro-Wilk test), therefore the Wilcoxon rank sum test was used for *post hoc* analysis. ***: P < 0.01, NS: not significant. (C) Axial patterning of embryos from mothers with *osk* genes as indicated. Mutant phenotypes, from mislocalized *osk* activity, are defined as in Fig 1 with the addition of bicaudal (3 or more duplicated abdominal segments). N values for all genotypes were greater than 300.

To explore these possibilities several additional mutants were tested (Fig 2). The first retained all features of *3*’Δ*550–597 tl* except for the tetraloop (*osk 3*’Δ*550–597 bl*; Fig 2A). Tetraloops stabilize or enhance folding of adjacent stem regions [20,21] and one was included in the *osk 3*’Δ*550–597 tl* mutant to compensate for a predicted reduction in folding stability due to the loss of the extended terminal stem region. Although the mutant without the tetraloop had hyperactivity phenotypes less extreme than for *osk 3*’Δ*550–597 tl*, anterior mislocalization of the mutant mRNAs was readily detected (Fig 2B) and all embryos were bicaudal (Fig 2C). Thus, *osk 3*’Δ*550–597 bl* substantially relieves inhibition of TAS activity independent of the presence of the tetraloop.

We next asked if bases flanking the TAS in SL2a and predicted to be unpaired (see Fig 2A) act to limit TAS activity. Some (*osk 3*’Δ*unp*) or all (*osk 3*’Δ*unpall*) of these were deleted with no other changes in SL2a (Fig 2A). Neither mutant mRNA was detectably mislocalized at the anterior (Fig 2B) but, in the sensitive embryonic body patterning assay, anterior defects were present in 1.0% (*osk 3*’Δ*unp*) or 3.3% (*osk 3*’Δ*unpall*) of the embryos, indicating a very low level of ectopic *osk* activity (Fig 2C).

Finally, we tested mutants that were similar to *osk 3*’Δ*550–597 tl* but removed less of the distal SL2a stem. Both *osk 3*’Δ*559–588 tl* and *osk 3*’Δ*563–583 tl* (Fig 2A) mRNAs were mislocalized at the anterior at low but significant levels (Fig 2B). Consistent with these results, in the body patterning assay anterior defects were present in 2.9% (*osk 3*’Δ*559–588 tl*) or 4.6% (*osk 3*’Δ*563–583 tl*) of the embryos (Fig 2C). Thus, the presence of a stable stem contributes to inhibition of TAS activity.

In conclusion, these results show that relief of TAS inhibition in *osk 3*’Δ*550–597 tl* cannot be wholly ascribed to a single type of change in SL2a. Instead, each recognizable type makes a contribution, and both of the two general options to explain the normally low activity of SL2a –inherently low affinity for transport/anchoring factors or binding of an inhibitory factor—remain viable hypotheses.

### EGL protein preferentially binds to active forms of the TAS

**Fig 3:**
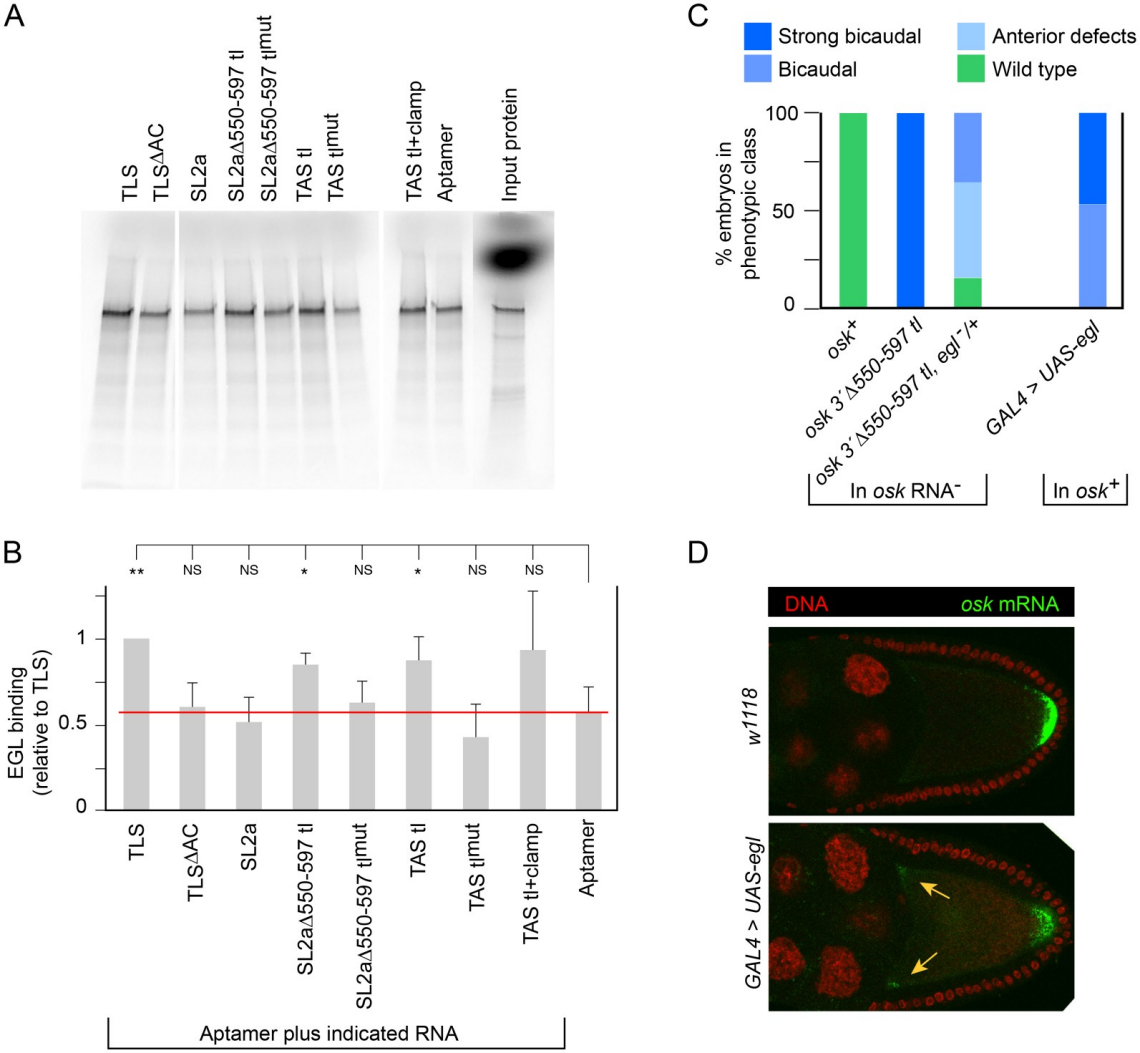
EGL binds the TAS *in vitro* and EGL level *in vivo* influences *osk* mRNA distribution and activity. (A) Representative binding assay. Radiolabeled EGL was synthesized in rabbit reticulocyte extracts for capture by RNAs tethered to streptavidin beads via the S1mx4 aptamer. Bound protein was visualized by SDS-PAGE and quantitated. The samples were from the same set of binding assays, analyzed on two separate gels. Input protein, not subjected to the binding assay, was 10% of that used in each binding assay (the diffuse spot of signal near the top of the ‘Input protein’ lane is the unincorporated ^35^S-methionine). RNAs are based on those shown in Fig 1D, with inactivating mutations corresponding to those within the TAS in mutant Δ550–597 tl 539–540 in Fig 1E. (B) Summary of three independent sets of binding assays. Results from each set of assays was standardized to the level of EGL binding to the TLS prior to analysis. Error bars show standard deviations. Statistical significance was evaluated by one-way ANOVA with F(8,18) = 4.44, P < 5 x 10^−3^. Normality could not be rejected (Bonferroni corrected Shapiro-Wilk test), therefore Student’s *t* test was used for *post hoc* analysis. **: P < 0.05, *: P < 0.1, NS: not significant. (C) Axial patterning of embryos from mothers with transgenes as indicated. The *egl*^-^/+ flies were heterozygous for *egl*^*1*^. GAL4 is the *matalpha4-GAL-VP16* driver. Mutant phenotypes, from mislocalized *osk* activity, are defined as in Fig 1 with the addition of bicaudal (more than 3 duplicated abdominal segments). N values for all genotypes were greater than 300 except for *GAL4 > UAS-egl*, which was 222. (D) Distribution of *osk* mRNA in late stage 9 egg chambers. Sites of abnormal *osk* mRNA enrichment in the oocyte for this stage are indicated with arrows (orange). 30 late stage 9 egg chambers of each genotype were scored for the presence of anterior *osk* mRNA by the approach described in Materials and methods. For *w*^*1118*^, 0/30 had signal at the anterior. For EGL overexpression, 24/30 had signal at one or both of the two anterior/lateral junctions in the focal plane. Statistical significance was evaluated by the Wilcoxon rank sum test with P < 2 x 10^−9^.

We synthesized EGL protein by coupled *in vitro* transcription and translation and tested its binding to RNA transport signals with an assay similar to one previously reported [14]. The RNAs were immobilized on streptavidin magnetic beads via a 3’ aptamer [22,23], and the aptamer alone was tested in parallel as a background control. EGL protein binds to the *fs (1)K10* TLS RNA, but binding is reduced when bulges in the stem loop are removed (TLS^ΔAC^) [14]. Consistent with this previous result, in our assays EGL binding to TLS^ΔAC^ was reduced to background levels (*i*.*e*., to the binding shown to aptamer alone; Fig 3A and 3B). TLS binding was, therefore, used as a reference point, and the results are expressed as a fraction of that value (Fig 3B).

Overall, EGL binding to SL2a, both wild type and variants, showed a good correlation with transport and anchoring activity. EGL did not bind intact SL2a above background. By contrast, EGL bound robustly to the Δ550–597 tl version of SL2a. Addition of the 539–540 mutation (in SL2aΔ550–597 tl^mut^), which disrupts TAS activity *in vivo* (see Fig 1A and 1B), reduced binding by EGL to background levels. The isolated TAS tl transport signal was also strongly bound by EGL, and addition of the 539–540 mutation (in TAS tl^mut^) reduced binding to background. EGL binding to the isolated TAS tl with an extended stem (TAS tl+clamp) was, on average, substantially higher than to the aptamer alone. Although the statistical analysis did not confirm this difference, the *in vivo* activity of this RNA suggests that the binding is meaningful.

Genetic tests provided further support for the notion that EGL mediates activity of the TAS. First, reducing *egl* gene dose lessened the effects of enhanced TAS activity of the *osk 3*’Δ*550–597 tl* mutant. Whereas the *osk 3*’Δ*550–597 tl* mutant in *egl*^+^ mothers produced all strongly bicaudal embryos, mutating one copy of *egl* resulted in a substantial shift towards wild type (Fig 3C). Second, increasing *egl* dose mimicked the effects of enhanced TAS activity. Expression of a *UAS-egl* transgene under control of the *GAL4*:*VP16-nos* driver is known to produce some bicaudal embryos, with *osk* mRNA inappropriately enriched in their anterior region [24]. To ask if the *osk* mRNA in ovaries with excess EGL was mislocalized in the same manner as from uninhibited TAS activity, we expressed *UAS-egl* using the *matalpha4-GAL-VP16* driver. In the ovaries, *osk* mRNA was retained at the anterior of late stage 9 egg chambers, much like the localization defect of the *osk 3*’Δ*550–597 tl* mutant (Fig 3D). All of the resulting embryos were bicaudal (Fig 3C).

Taken together, our *in vitro* and *in vivo* results are consistent with a role for EGL in transport and anchoring of *osk* mRNA at the oocyte anterior via binding to the TAS.

### The predicted SRSs in the *osk* 3’ UTR bind to STAU

A candidate to inhibit TAS activity in the context of SL2a is STAU. Notably, SL2a includes two predicted binding regions for STAU [19], where one is removed in the *osk 3*’ Δ*550–597 tl* mutant in which we have shown that inhibition of TAS activity is alleviated.

**Fig 4:**
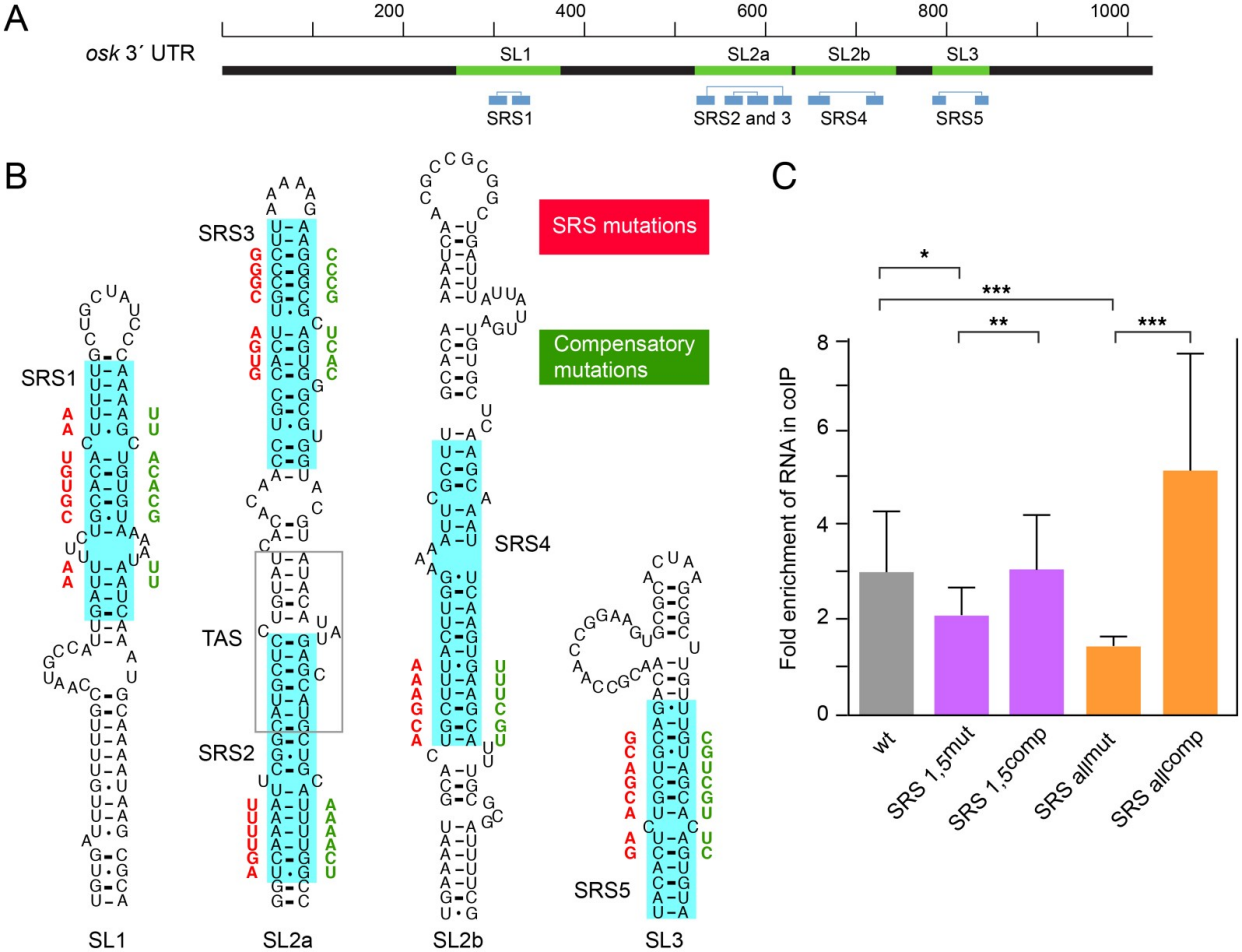
The predicted SRSs in *osk* mRNA mediate STAU binding. (A) Schematic of the *osk* mRNA 3’ UTR showing predicted stem-loop regions (green) and the position of SRSs [19]. The two strands of each SRS duplex region (blue) are connected by thin lines above. (B) Predicted structures of the stem-loop regions with SRSs highlighted in blue. Mutations to disrupt SRSs are shown in red, with compensatory changes in green. The TAS is outlined in grey. (C) Enrichment of *osk* 3’ UTR RNAs in anti-STAU coimmunoprecipitates. Values are from at least 4 biological replicate assays for each 3’ UTR shown on the x-axis. To calculate fold enrichment as determined by RT-qPCR (y-axis), Firefly *luciferase-osk 3’ UTR* expression was normalized, first to *Renilla luciferase* expression from the IP, then to input, and finally to Firefly *luciferase*-only vector. Error bars indicate standard deviation. Statistical significance was evaluated by one-way ANOVA with F(4,31) = 6.68, P < 6 x 10^−4^. Normality could not be rejected (Bonferroni corrected Shapiro-Wilk test), therefore Student’s one-tailed *t* test was used for *post hoc* analysis. ***: P < 0.01, **: P < 0.05, *: P < 0.1.

To define presumptive STAU binding sites, Laver et al. [19] used an RNA coimmunoprecipitation (RIP) strategy, isolating mRNAs associated with STAU in *Drosophila* embryos and then carrying out a computational search for features shared by bound transcripts relative to co-expressed unbound transcripts. They identified several classes of STAU Recognized Structures (SRSs), which consist of variants of duplex regions within stem loop structures. Multiple SRSs are predicted in the *osk* 3’ UTR [19], and all are overlapping versions of what we call SRSs 1–5, which reside in four stem loop structures (SL1, SL2a, SL2b and SL3; Fig 4A and 4B). As a prelude to considering the possible role of STAU in modulating TAS activity, we assessed whether SRSs are *bona fide* STAU binding sites, using the *osk* mRNA as a test case. Our approach was to mutate the SRSs, first altering bases in one strand of the duplex to disrupt an SRS, then modifying the initial mutant with compensatory changes to restore the duplex and the SRS (but now with a different sequence; Fig 4B). With these pairs of mutants, a RIP assay was used to test for STAU binding, and *osk* transgenes were used to ask if they recapitulate the effects of *stau* mutants on *osk* regulation *in vivo*.

For the RIP binding assay, versions of *osk* mRNA 3’ UTRs (Fig 4B) were fused to the Firefly *luciferase* open reading frame, and then expressed from DNAs transfected into *Drosophila* S2 cells (*Renilla luciferase* was used as a transfection control) together with 3xFLAG-tagged STAU. For the reason addressed below, the *osk* 3’ UTRs had all SRSs mutated, or just SRSs 1 and 5. After RIP with FLAG-STAU, co-IPed RNA levels were measured by RT-qPCR and double normalized as described in the Materials and methods. The results are shown in Fig 4C. Wild-type *osk* 3’ UTR was about three-fold enriched in the co-IPed RNA. Mutation of SRSs 1 and 5 significantly reduced STAU binding, while mutations of all SRSs together showed the greatest reduction. Inclusion of compensatory changes in the SRSs to restore duplex formation also restored binding to STAU. These results argue that the SRSs predicted in the *osk* 3’ UTR are *bona fide* STAU binding sites.

### STAU-independent consequences of mutating SRSs

Next, the above SRS mutations were introduced into *osk* transgenes for testing in the *osk* RNA-null background. A primary goal was to ask if reduced STAU binding mimics the effects of *stau* mutants on *osk* mRNA regulation, thereby confirming that the SRSs mediate the action of STAU on *osk* mRNA. In addition, we wanted to ask if SRS mutations in SL2a could be used to test the model that STAU binding close to the TAS inhibits its activity. The answer to the latter question was no, and we address the underlying cause first because it informs the interpretation of SRS mutant phenotypes.

**Fig 5:**
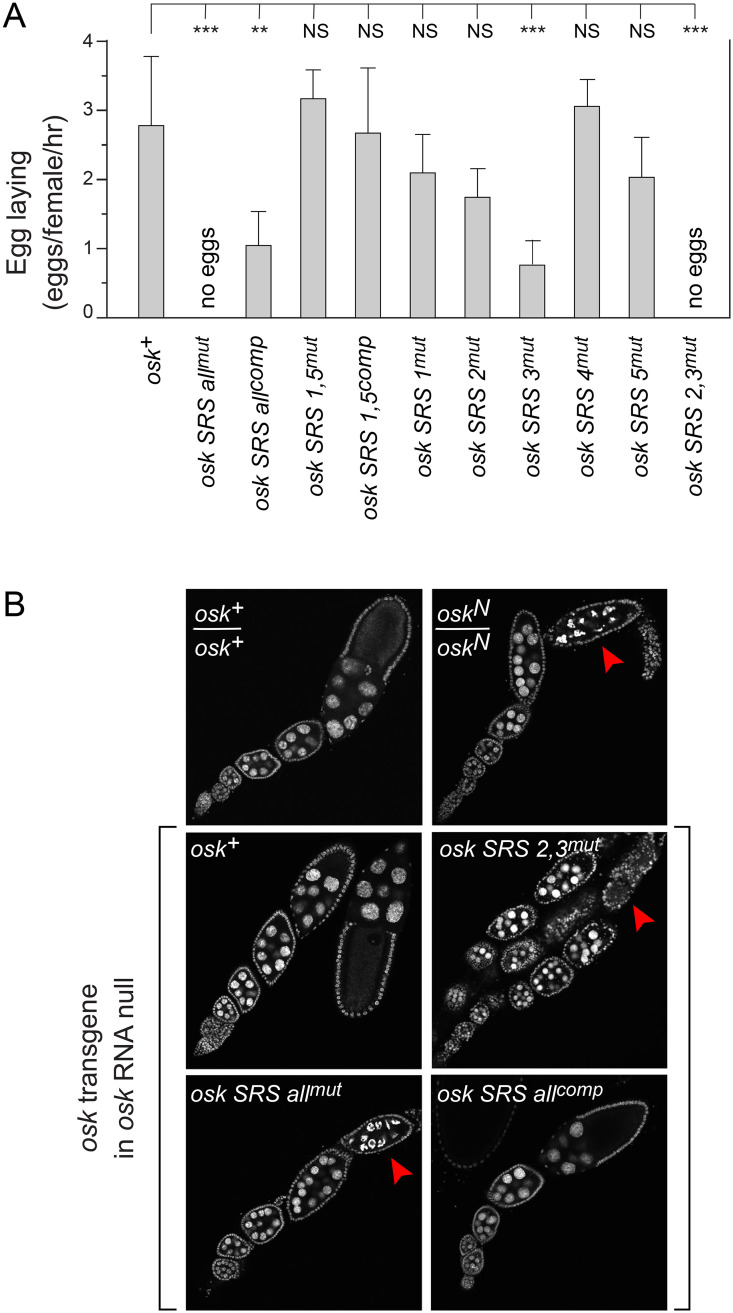
STAU-independent effects of SRS mutations. (A) Egg laying assays to monitor effects of SRS mutations on progression through oogenesis. Error bars show standard deviations. Statistical significance was evaluated by one-way ANOVA with F(10,38) = 19.26, P < 6 x 10^−12^. Normality was rejected (Bonferroni corrected Shapiro-Wilk test), therefore the Wilcoxon rank sum test was used for *post hoc* analysis. ***: P < 0.01, **: P < 0.05, NS: not significant. (B) Timing of arrest of oogenesis from SRS mutations. Each panel shows one or more ovarioles from a female of the indicated genotype for *osk*, stained with ToPro-3 to reveal DNA (nuclei). Arrest of oogenesis results in dying egg chambers, indicated with red arrowheads. The stage of arrest from the SRS mutations was similar to that of the *osk* RNA null mutant, *osk*^*N*^. A phenotype frequently observed in *osk* RNA null mutant ovarioles, supernumerary nurse cells, was also found in the SRS mutants causing arrest of oogenesis. The SRS compensatory mutations, in *osk SRS all*^*comp*^, restore both progression through oogenesis and the correct number of nurse cells.

When all SRSs in *osk* mRNA were mutated (*osk SRS all*^*mut*^), no eggs were laid (Fig 5A); this is not a phenotype of *stau* mutants [25] and so at least some SRS mutations must have effects not attributable to loss of STAU binding. Most egg chambers of *osk SRS all*^*mut*^ failed to progress beyond stage 8 or early stage 9 (Fig 5B). The arrest was rescued by compensatory mutations (in *osk SRS all*^*comp*^)(Fig 5B), with a substantial increase in egg laying (Fig 5A).

The arrest of oogenesis from mutation of all SRSs is readily explained. Although the SRS mutations did not change the sequence of the TAS in SL2a (Fig 4B), loss of base pairing in both proximal and distal portions of the SL2a stem could alter proper folding of the signal. TAS activity is required for *osk* ncRNA function [11], and disruption of *osk* ncRNA function results in arrest of oogenesis [6]. RNA folding predictions support this interpretation: considering folding of the entire SL2 region, when SRSs 2 and 3 were both mutant (in the *osk SRS all*^*mut*^ transgene) the TAS (*i*.*e*., the consensus structure of S1 Fig) did not appear in any predicted fold whose ΔG was within 10% of the most stable version (S2 Fig).

To further explore effects of SRS mutants on STAU-independent disruption of oogenesis, we tested additional mutants. The outer SRSs, 1 and 5, lie within predicted stem loop structures not previously implicated in any aspect of *osk* mRNA regulation or function, and mutation of both had no effect on egg laying (Fig 5A).

Testing transgenes with individual SRSs mutated reinforced the conclusion that arrested oogenesis for the *osk SRS all*^*mut*^ transgene came from changes within SL2a. Only from mutation of SRS 3 (in SL2a) was there a significant reduction in rate of egg laying (Fig 5A). For the SRS 3 mutant, the overall folding stability of SL2 was reduced, and the TAS structure was not necessarily present in the most stable fold (S2 Fig). Combining both SRS 2 and 3 mutations (in *osk SRS 2*,*3*^*mut*^) arrested oogenesis (Fig 5B) and eliminated egg laying (Fig 5A). Consistent with these defects, folding predictions showed loss of the TAS structure (S2 Fig).

SRS 4 lies within SL2b, the location of the oocyte entry signal (OES) [10], which is also required for *osk* ncRNA activity [7,11]. Based on the properties of deletion mutants in SL2b [11], the SRS 4 mutation would not be predicted to have a substantial effect on the OES contribution to *osk* ncRNA activity. Furthermore, even suboptimal folds with the SRS 4 mutant did not affect formation of the TAS structure in SL2a (S2 Fig). Consistent with the absence of any substantial predicted effect on either TAS folding or OES activity, egg laying remained strong for the SRS 4 mutant (Fig 5A).

Disruption of TAS activity from mutation of SRSs 2 and 3 ruled out use of these mutations to test any model for how TAS activity might be inhibited. It is important to emphasize that the defects of SRS 2 and 3 mutants can be fully explained by indirect effects on TAS folding, and there is no reason to believe that STAU binding is required for TAS activity; we argue below that STAU binding inhibits TAS activity.

### SRS phenotypes that mimic the *stau* mutant phenotype

**Fig 6:**
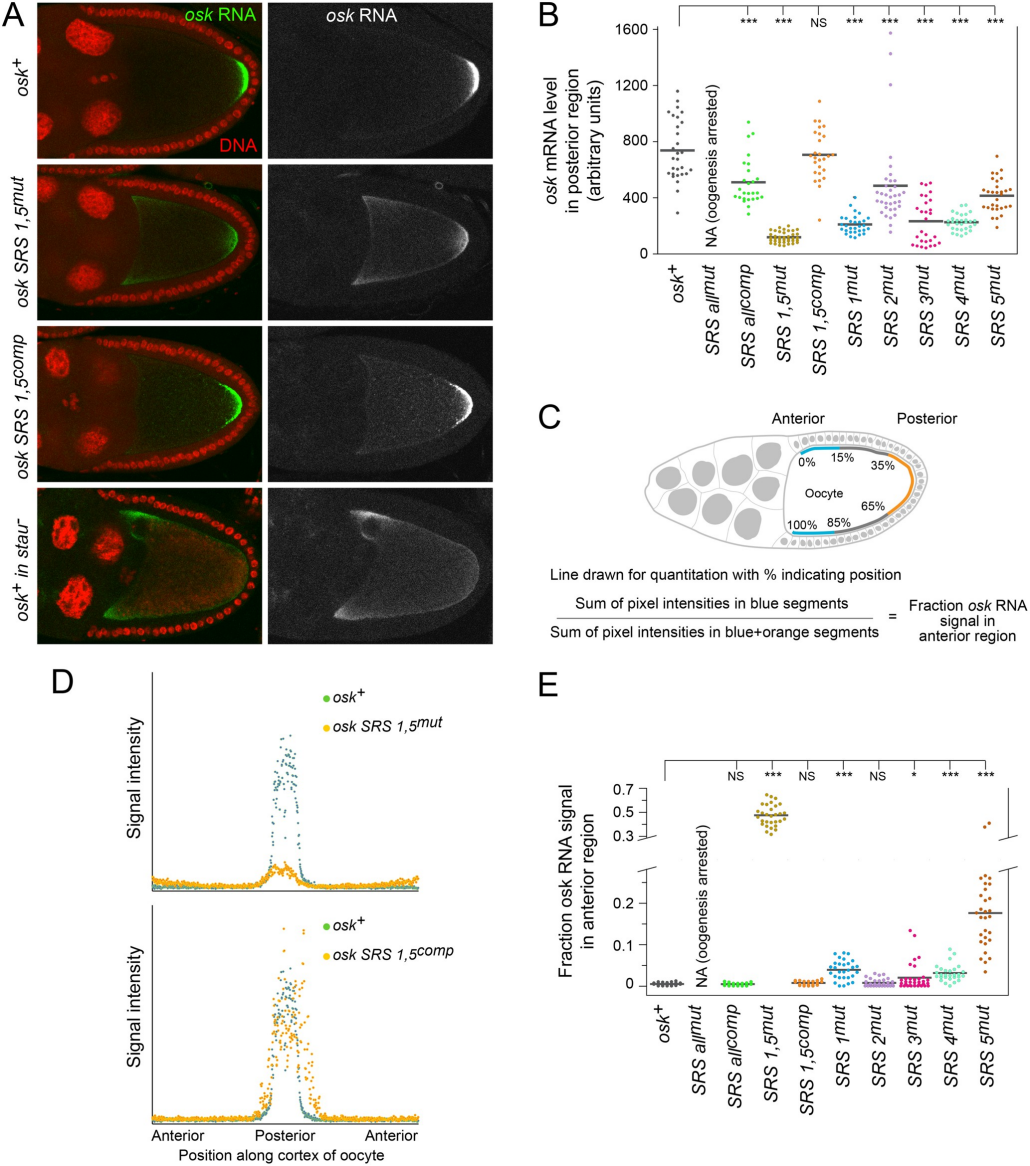
Characterization of genomic *osk* transgenes carrying SRS mutations. (A) Distribution of *osk* mRNAs in late stage 9 egg chambers. For the right panels, only the RNA channel is shown (white). The bottom panels are not directly comparable for signal intensity (2 copies of endogenous *osk* for the bottom panel vs 1 copy of *osk* transgenes for the upper three panels, and different imaging sessions) but can be compared for mRNA distributions. (B) Posterior localization of *osk* mRNAs. Values are for total signal intensity within the posterior region of localization, not solely at the cortex as in panel D. Results are presented in dot plot format. N values for all genotypes were 26 or greater. Statistical significance was evaluated by one-way ANOVA with F(8,264) = 53.52, P < 6 x 10^−51^. Normality was rejected (Bonferroni corrected Shapiro-Wilk test), therefore the Wilcoxon rank sum test was used for *post hoc* analysis. ***: P < 0.01, NS: not significant. (C) Method for quantitation of fraction of *osk* mRNA signal in anterior region. Signal intensities were measured at multiple points in lines traced along the cortex, starting at the anterior edge, through the posterior, and back to the anterior edge on the other side. (D) Comparison of mRNA distributions from quantitation, shown schematically in panel C, of specific images in panel A. (E) Quantitation of anterior retention of *osk* mRNAs, obtained as in panels C and D. Values, presented in dot plot format, indicate the fraction of signal from the most anterior 15% on each side of the oocyte, relative to the sum of this anterior fraction plus the signal from the central 30% of the trace (i.e. the posterior region) (schematic in panel C). N values for all genotypes were 17 or greater. Statistical significance was evaluated by one-way ANOVA with F(8,242) = 329.45, P < 2 x 10^−125^. Normality was rejected (Bonferroni corrected Shapiro-Wilk test), therefore the Wilcoxon rank sum test was used for *post hoc* analysis. ***: P < 0.01, *: P < 0.1, NS: not significant.

SRS mutants that did not strongly disrupt TAS activity could be tested for effects on *osk* mRNA distribution in the later stages of oogenesis, and thus could be compared to the effect of mutation of *stau*. In *stau* mutants the level of *osk* mRNA at the posterior of the oocyte is greatly reduced, and there is significant retention of the mRNA at the anterior of the oocyte [16–18] (Fig 6A). We measured both posterior localization and degree of anterior retention. Mutation of SRSs 1 and 5 together caused a significant reduction in posterior localization of *osk* mRNA within stage 9 and 10A oocytes (Fig 6A and 6B), with substantial levels concentrated in cortical regions at or near the anterior (Fig 6A, 6D and 6E; the strategy for anterior RNA quantitation is shown in Fig 6C). Parallels with the *stau* mutant phenotype extended to loss of posterior pattern elements in embryos (S3 Fig) and reduced OSK protein accumulation (S4 Fig). Each of these defects was rescued by compensatory mutations in SRSs 1 and 5 (Fig 6A, 6B, 6D, 6E, S3 and S4 Figs). Similarly, the transgene in which all SRSs were mutated together with compensatory changes had significant posterior localization of the mRNA and close to wild-type embryonic body patterning (Fig 6B and 6E and S3 Fig).

Mutation of any individual SRS also substantially reduced the amount of *osk* mRNA localized to the posterior pole of the oocyte (Fig 6B) and interfered with body patterning (S3 Fig). For mutants of SRSs 1, 4 or 5, the reduction in posterior localization was accompanied by anterior retention of the mRNAs (Fig 6E), as would be expected based on the observed *stau* mutant phenotype. By contrast, there was no anterior enrichment for mutants of SRSs 2 or 3. This could reflect some diversification in SRS function, but is more simply explained by reduced anchoring since it is mutation of these SRSs that interfered with folding of the TAS (S2 Fig), and any reduction in TAS cortical anchoring activity would reduce the degree of anterior retention.

The striking parallels between SRS mutant and *stau* mutant phenotypes reinforce the conclusion that the *osk* mRNA SRSs—all of them—mediate the action of STAU, although not necessarily all by the same mechanism. The position of two SRSs closely flanking and overlapping with the TAS (see Fig 4B) supports the notion that one mode of STAU action may be to regulate TAS activity.

### STAU inhibits activity of SL2a in both RNA transport and cortical anchoring

Although the SRS mutants *per se* were not useful to test the model that STAU exerts an inhibitory effect on TAS activity, we could instead ask if TAS activity is affected in *stau* mutant ovaries. Notably, STAU is highly concentrated in the oocyte, with only low levels detectable in nurse cells [25]. Consequently, STAU might be expected to have a more limited effect on the process occurring primarily within the nurse cells (*i*.*e*., transport to the oocyte) and a stronger influence on cortical anchoring within the oocyte.

**Fig 7:**
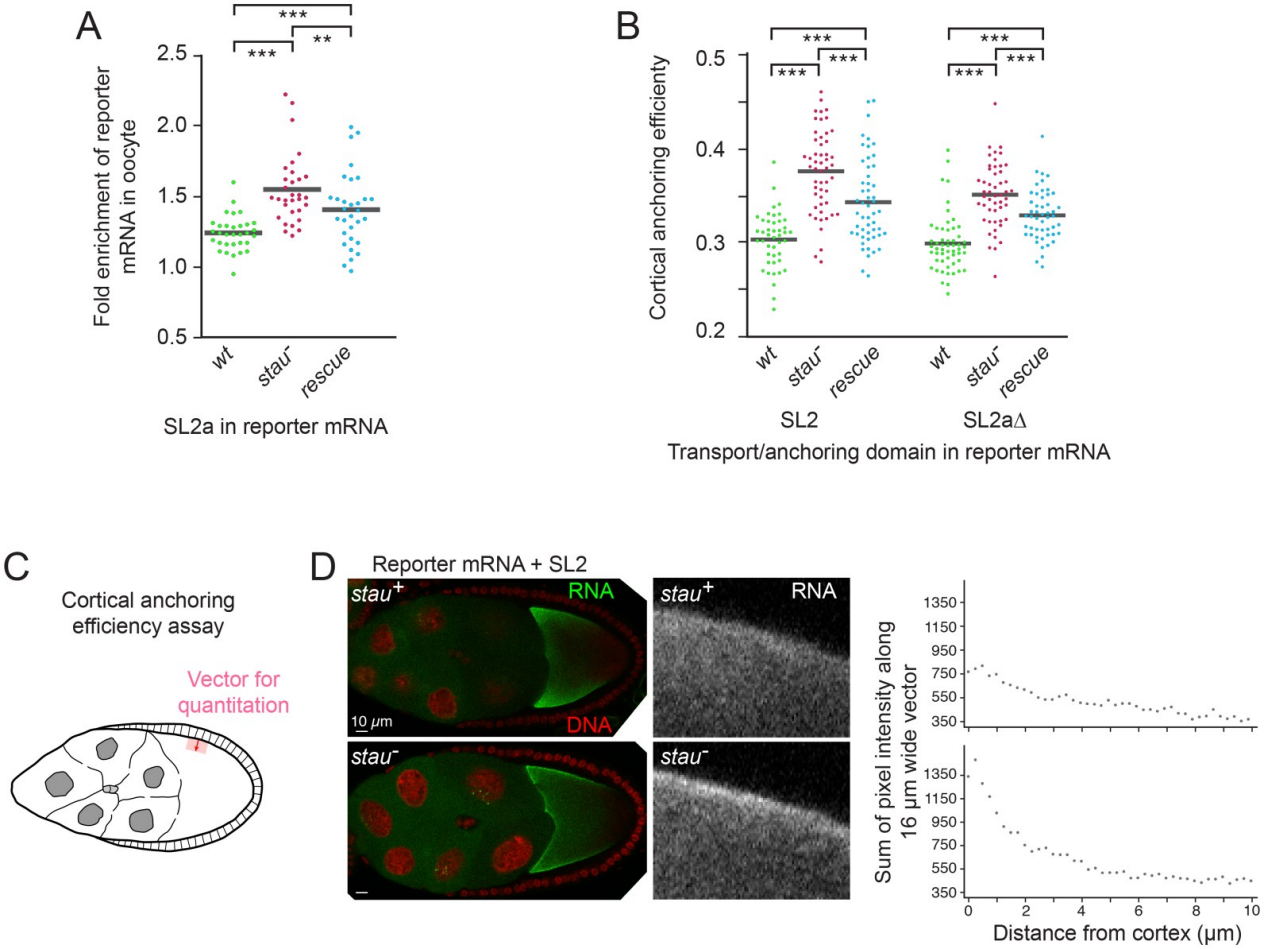
STAU inhibits TAS transport and cortical anchoring activities. Reporter mRNAs with the SL2a domain of *osk* (panel A) or forms of the TAS (panel B) were expressed in wild type (wt), homozygous *stau*^*C*^ mutant (*stau*^-^) or homozygous *stau*^*C*^ mutant with a *stau*^+^ transgene (rescue) ovaries. (A) Efficiency of RNA transport to the oocyte was measured by comparing reporter mRNA levels in nurse cells and oocyte of individual stage 4–6 egg chambers (Materials and methods). Transport was elevated in the absence of *stau*, and this effect was substantially reversed by the *stau*^+^ transgene. The failure to completely reverse the elevated transport (as well as the elevated cortical anchoring in panel B) may be due to differences in *stau* gene dosage, as the rescuing transgene is present in only one copy, or could be due to lower expression from the transgene relative to the endogenous *stau* gene. Results are presented in dot plot format. N values were at least 31 for all genotypes. Statistical significance was evaluated by one-way ANOVA with F(2,91) = 15.52, P < 2 x 10^−6^. Normality was rejected (Bonferroni corrected Shapiro-Wilk test), therefore the Wilcoxon rank sum test was used for *post hoc* analysis. ***: P < 0.01, **: P < 0.05. (B) Efficiency of RNA cortical anchoring was measured in early stage 9 egg chambers using the approach outlined in panel C. Both forms of SL2a showed enhanced anchoring in the absence of STAU, and this effect was significantly reversed by the *stau*^+^ transgene. Results are presented in dot plot format. N values were at least 44 for all genotypes. Statistical significance was evaluated by one-way ANOVA with F(5,296) = 33.90, P < 3 x 10^−27^. Normality was rejected (Bonferroni corrected Shapiro-Wilk test), therefore the Wilcoxon rank sum test was used for *post hoc* analysis. ***: P < 0.01. (C) Schematic diagram of the approach to measure cortical anchoring efficiency. A 10 μm vector (dark red line with arrowhead) was drawn from the lateral cortex towards the interior of the oocyte. At intervals along the vector, but 16 μm wide (red shaded area), signal intensities were measured. Cortical efficiency was calculated as the proportion of signal in the first 2.5 μm relative to the summed signal along the entire vector. (D) Examples of egg chambers scored for cortical anchoring efficiency, in the wild-type background at top and the *stau* mutant background at bottom. At left are the egg chambers showing the distribution of the reporter mRNA + SL2, with magnified portions of the cortical region and only the RNA signal at center. At right are the pixel intensities along the vector as measured in the assay using the same egg chambers.

To test the effects of loss of STAU on RNA transport we took advantage of the low transport activity of isolated SL2a, which simplifies detection of even a small increase. The reporter transgene with SL2a was compared for oocyte enrichment in wild-type and *stau* mutant backgrounds. Transport into the oocyte was improved in homozygous *stau* mutant ovaries, and inclusion of a rescuing *stau*^+^ transgene significantly reversed this effect (Fig 7A).

To test the effects of loss of STAU on cortical anchoring, an active TAS is required. Two versions of SL2a were used. One had SL2a together with SL2b (in SL2) and thus contains SRSs 2–4. The second had SL2a in the Δ550–597 tl form (see Fig 1A) to enhance transport and anchoring activity. This version retains SRS 2 in the proximal stem.

As a measure of cortical anchoring, RNA intensity was measured along vectors 10 μm long and 16 μm wide extending from the anterior lateral cortex into the interior of the oocyte (Fig 7C). Representative images and traces obtained from them are shown in Fig 7D. The fraction of RNA in the most cortical 2.5 μm, relative to the sum of RNA along the 10 μm vector, serves as a measure of cortical anchoring efficiency. For both forms of SL2a, the cortical anchoring efficiency increased significantly in the *stau* mutant (Fig 7B), consistent with the more prominent cortical concentration visible in the images (Fig 7D). Just as observed for transport activity, addition of a *stau*^+^ transgene significantly reversed the effect of the *stau* mutation (Fig 7B). Taken together these results lead us to conclude that STAU has an inhibitory effect on the anchoring activity of the TAS.

## Discussion

We have shown that EGL binds to the TAS element of the *osk* mRNA’s 3’ UTR. Just as for the *fs(1)K10* TLS paradigm of a regulatory element bound by EGL, active forms of the TAS mediate not only transport to the oocyte but also cortical anchoring once inside the oocyte. Previous work is conflicted on the role of the EGL/BICD system for transport of *osk* mRNA from the nurse cells to the oocyte. Coimmunoprecipitation studies have shown association of EGL with *osk* mRNA [26], and certain mutations in *BicD* alter the localization of *osk* mRNA [17]. However, *osk* differs from multiple mRNAs transported by the EGL/BICD system in failing to undergo strong apical localization when injected into early stage embryos [10,13], a difference that can now be understood (below).

The minimal TAS is active and is bound by EGL. However, in the context of the isolated SL2a stem loop domain (as opposed to the complete *osk* mRNA), the TAS has little transport and anchoring activity and is not bound by EGL above background. We previously proposed that weak association of transport factors with the signal in SL2a would facilitate transfer of the mRNA to other factors for the subsequent step of posterior localization [11]. Now, with evidence that the TAS mediates both transport and anchoring, the notion of a need to modulate TAS activity becomes more compelling, as persistent anchoring would unquestionably interfere with posterior localization.

Evidence that TAS activity is regulated came from the discovery that an *osk* mutant mRNA is mislocalized in a manner much like an *osk* mRNA to which the TLS was added. Because the TAS itself was not altered, the mutant appeared to be defective in an inhibitory effect on TAS activity. Experiments detailed here with reporter transgenes comparing wild-type and mutant versions of SL2a have confirmed that the latter is relieved from inhibition of TAS activity, for both transport and anchoring. Efforts to assign the inhibitory effect to a specific feature of SL2a have shown that no single difference is solely responsible; instead, the several differences in the mutant have effects. One of these differences, the deletion of a terminal region of the SL2a stem, results in loss of one of two predicted binding sites in SL2a for STAU, a protein implicated in *osk* mRNA localization.

The presence of predicted STAU binding sites, SRSs, in close proximity to or overlapping with the TAS in SL2a, suggested a possible role for STAU in regulating TAS activity. To explore this possibility we needed first to determine if the predicted SRSs [19] are *bona fide* STAU binding sites. Binding studies with *osk* RNAs with altered SRSs, and analysis of the behavior of the same mutants *in vivo*, validated STAU binding. While in this study we have only tested SRSs in the *osk* mRNA, validation of the STAU binding site predictions raises confidence in their use for analysis of the extent and diversity of STAU function.

Mutation of SRSs that are distant from the stem loops acting in the transport of *osk* mRNA to the oocyte mimicked the effect of *stau* mutations on *osk* mRNA localization. There was no decrease in transport of the mRNA to the oocyte (S5 Fig), but within the oocyte, localization to the posterior was substantially reduced and a fraction of the mRNA remained at anterior and cortical regions. These defects, rescued by compensatory changes that restore RNA duplexes that comprise SRSs, represent a weaker version of how *osk* mRNA is affected in *stau* mutant ovaries, as might be expected for partial loss of STAU binding to the mRNA. Furthermore, the reduction in posterior localization from mutation of SRSs not implicated in regulating transport or anchoring signals (and not close to them in the primary RNA sequence) is consistent with a model in which STAU plays a role in facilitating directed movement of the mRNA to the posterior pole [25].

We were unable to use *osk* mRNAs with altered SRSs to evaluate a possible additional role for STAU in regulating TAS activity: the mutations are predicted to destabilize the SL2a structure, including folding of the TAS. Instead, we asked if absence of STAU would relieve inhibition of TAS activity, which is normally strong in the context of the complete SL2a fused to a reporter mRNA. Use of the reporter mRNA eliminated the complication, when testing *osk* mRNA, of being unable to distinguish between two events: release from anchoring at the anterior cortical regions, and directed posterior localization, which the reporter mRNA does not undergo. We found that STAU does contribute to inhibition of TAS activity, both in transport and in cortical anchoring. How STAU inhibits TAS activity is unknown, but with two SRSs near to or overlapping the TAS, an inhibitory effect on EGL binding is plausible. Demonstration of a specific molecular mechanism may emerge from biochemical reconstructions with purified STAU and EGL, which are beyond the scope of this report.

Evidence that STAU inhibits TAS anchoring activity supports the notion that STAU plays two roles in *osk* mRNA localization. In addition to a possibly direct role in posterior movement, consistent with the behavior of *osk* mRNA with mutations in SRSs 1 or 5, STAU also acts to remove or relax the tension between competing machineries: the anchoring machinery working to hold the mRNA in place at the anterior where it entered the oocyte, and the posterior localization machinery working to move the mRNA to the posterior of the oocyte. The effect of *stau* mutants on localization of endogenous *osk* mRNA—anterior retention and loss of posterior localization—is concordant with both roles. A related version of a role for STAU in releasing *osk* mRNA from the transport machinery was suggested previously [25] in the context of explaining why reducing *stau* activity enhances the defects of the dominant *BicD*^*1*^ mutant [17,25]. Biochemical properties of BICD and of the *BicD*^*1*^ mutant, obtained more recently, are illuminating [24,27–29]. BICD acts to link a cargo—in this case EGL and associated RNA—to Dynein for transport along microtubules. BICD binding to Dynein is autoinhibited by interaction between the coiled coil domains CC3 and CC2/CC1 of BICD. Cargo binding to CC3 causes a rearrangement of CC3, altering its interaction with CC2/CC1 which becomes available for Dynein binding. The *BicD*^*1*^ mutation appears to disrupt heterotypic core packing of the CCs, removing the autoinhibitory interaction and promoting Dynein binding, but only when cargo is bound [24]. Thus, in *BicD*^*1*^ mutant ovaries the EGL/*osk* mRNA cargo is too consistently bound to the transport and anchoring machinery, interfering with the handoff to the posterior localization machinery. If STAU acts to displace the transport machinery, as we suggest, then reducing the level of STAU will exacerbate the *BicD*^*1*^ phenotype, as observed [25].

Inhibition of TAS activity by STAU may be concentration-dependent: loss of STAU has a modest effect on transport from nurse cells, where STAU is present at very low levels, while loss of STAU has a greater effect on anchoring in the oocyte, where STAU is abundant. This interpretation explains the weak apical localization of *osk* RNA in early embryos, relative to other RNAs with stem loop structures bound, or potentially bound, by EGL [10,13]. In early embryos, STAU, while enriched at anterior and posterior poles, is present at significant levels throughout [25]. Thus, STAU could bind to *osk* RNA throughout the embryo and inhibit its apical transport and its anchoring.

Although our evidence supports the model of STAU acting to inhibit the activity of the TAS, two observations argue that this is not the only means by which the TAS is regulated. The changes in *osk 3*’Δ*550–597 tl* substantially alleviate inhibition of TAS activity. While this effect may be due in part to the loss of SRS 3 in the distal stem of SL2a, smaller deletions that also disrupt SRS 3 (*e*.*g*., *osk 3*’Δ*559–588 tl*) did not have such a dramatic effect on TAS activity. Thus, the larger deletion appears to relieve inhibition of TAS activity in more than one way. Second, removing STAU only modestly improves transport activity of the TAS in the context of SL2a, while the minimal, uninhibited forms of the TAS have higher transport activity. It is notable that the combined SL2a and SL2b, in SL2, provide robust transport, well beyond the low activities of either stem-loop alone and suggesting a synergistic effect. It would not be surprising if interplay between undefined regulatory elements in the different stem loops serves both to enhance transport and to inhibit anchoring.

EGL binds preferentially to RNA duplexes that adopt the A’ form, which facilitates minor groove contacts [14,30]. STAU is predicted to bind to duplexes with a small number of non-Watson-Crick and unpaired bases (*i*.*e*., bulges) [19] and a recent structural analysis of human STAU1 binding to part of the *Arf1* mRNA suggests specific base contacts via the minor groove [31]. Because both proteins bind duplex RNA, overlap in their binding sites as found in SL2a would not be unusual. Perhaps STAU proteins act by modulating the activity of other proteins that bind duplex RNA, with EGL being only one example of this phenomenon. Such a role would help explain the wide variety of post-transcriptional control mediated by STAU proteins, including transport, translation and decay of mRNAs, as well as modulation of microRNA activity [18,32–35].

## Materials and methods

### Flies and transgenes

Genomic *osk* transgenes were based on a genomic fragment that fully rescues *osk* null mutants [16] and made by phiC31 transgenesis to the attP site on chromosome II at 51D (in Bloomington stock center strain 24483). Those from prior work were inserted on chromosome II at 51C (in Bloomington stock center strain 24482), which provides a similar level of expression. The genomic *osk* transgenes were tested in an *osk* RNA null combination, typically *osk*^*N*^ homozygotes but in some cases using *Df(3R)osk* [36] or *osk*^*0*^ [7] alleles. The *osk*^*N*^ allele was generated by CRISPR/Cas9 methodology, and carries a deletion removing much of the *osk* gene (positions 3R:8,936,316–8,967,424 in r6.34). All *Drosophila* genomic sequence coordinates were obtained from FlyBase [37].

The *stau*^*C*^ mutant was generated by CRISPR/Cas9 methodology, and carries a deletion removing almost the entire coding region (positions 2R:18,119,804–18,124,777 of r6.36 of the genome sequence). The rescuing *stau*^+^ transgene included genomic sequences positions 2R:18,116,718–18,128,607, r6.36.

Reporter GFP transgenes are derivatives of *UAS-osk*::*GFP*, which includes a 5’ portion of the *osk* gene and coding region (*osk* mRNA coordinates 1–534, including the first 173 amino acids of Long Osk) [38]. The *matalpha4-GAL-VP16* driver [39] inserted on chromosome 3 (Bloomington *Drosophila* stock center stock #7063) was used for expression of *UAS* transgenes, all made by P element transgenesis.

The *UAS-egl* transgene was from Helen Salter and Simon Bullock [24] and was used in combination with the *matalpha4-GAL-VP16* driver.

Mutations or additions to transgenes were made using synthetic DNA fragments (gBlocks; Integrated DNA Technologies).

Egg laying assays were performed as described [11].

### Detection of RNAs and proteins

RNA levels were determined by RT-qPCR. RNA was extracted from whole ovaries using the mirVana isolation kit (Ambion/Thermo Fisher Scientific). Reverse transcription using 1 μg RNA was performed with Sensifast cDNA kit (Bioline) with random hexamer and oligo d(T) primers following the manufacturer’s protocol. qPCR on cDNA samples was performed in triplicate on ViiA7 (Applied Biosystems/TF) with Sensifast SYBR LoRox qPCR Supermix according to the manufacturer’s protocol for two-step cycling with 40 cycles and a 25 second annealing/elongation step for both *osk* and *RpL32* mRNAs. Primers with comparable lengths, melt temperatures and G/C content were selected from the FlyPrimerBank database (https://www.flyrnai.org/flyprimerbank) (*osk*: ATGACCATCATCGAGAGCAACT and GTGGCTCAGCAATATGGCG; *RpL32*: GCCCAAGGGTATCGACAACA and GCGCTTGTTCGATCCGTAAC). A five-log-order standard series of a control *osk* transgene with wild-type activity in an *osk* RNA null background served as a control for copy number and for plate reaction efficiency. A comparable dilution series was made by pooling all samples to be run on a plate, and the efficiency was compared to that of the standard series. All efficiencies were between 90–110%, and efficiencies from the sample pool were within 5% of the standard series. Cycles to threshold were calculated in QuantStudio (Thermo Fisher Scientific), and the RNA level of *osk* relative to *RpL32* for each sample was normalized to respective *osk*^+^ transgene controls with matched efficiency by the 2^-ΔCt method, dividing the average of sample biological replicates by that of the control [40]. At least three biological samples were tested for each mutant, except *osk 3’ unp* and *osk 3’ unpall*, for which one sample was dramatically different from all the others tested and so was excluded. Melt curves were screened for multiple species and consistent melt temperatures, and technical replicates with a deviation of more than 0.5 cycles to threshold were rejected. RNA levels for the transgenes first used in this report are presented in Table 1. There are three instances in which the levels differed from the *osk*^+^ control by more than two-fold: *osk 3*’ Δ*550–597 tl*, *osk 3*’ Δ*550–597 bl* and *osk 3’ unpall*, with each at higher levels than the *osk*^+^ control. Although elevated levels of the first two of these mutant mRNAs may have contributed to the severity of their body patterning defects, the changes in mRNA distribution (the phenotype most relevant to the conclusions) cannot be attributed to higher levels. The transgenes were present in single copies (as was the *osk*^+^ control), and their levels were only slightly above that of endogenous *osk* in wild-type ovaries where two copies of *osk* are present. Moreover, increasing the dosage of wild-type *osk* to substantially higher levels does not result in similar anterior accumulation of *osk* mRNA [5]. Most importantly, the key conclusion from these transgenes, that the cortical anchoring activity of the TAS in SL2a is normally subject to inhibition, was confirmed by experiments with reporter transgenes. For the third mutant mRNA present at the higher level (but effectively the same as *osk*^+^ in wild-type flies), *osk 3’ unpall*, there was no observed mislocalization of the mRNA. Any effect of the mutation on causing inappropriate expression of OSK protein would have been enhanced by higher mRNA levels yet there was only a very low level of ectopic *osk* activity.

**Table 1:** Relative levels of genomic *osk* transgene mRNAs.

Transgene	mRNA level^a^	Standard deviation
*osk*^+^	1.00	
*osk 3*’ Δ*550–597 tl*	2.47	1.79
*osk 3*’ Δ*550–597 tl 539–540*	0.93	0.44
*osk 3’ 539–540*	1.02	0.48
*osk 3*’ Δ*550–597 bl*	2.07	2.07
*osk 3*’ Δ*559–588 tl*	0.84	0.09
*osk 3*’ Δ*563–583 tl*	1.60	0.75
*osk 3*’ Δ*unp*	1.73	1.52
*osk 3*’ Δ*unpall*	2.04	0.44
*osk SRS all*^*mut*^	1.37	0.73
*osk SRS all*^*comp*^	0.84	0.13
*osk SRS 1*,*5*^*mut*^	1.71	1.12
*osk SRS 1*,*5*^*comp*^	1.58	1.14
*osk SRS 1*^*mut*^	1.67	0.98
*osk SRS 2*^*mut*^	1.53	0.99
*osk SRS 3*^*mut*^	1.26	0.42
*osk SRS 4*^*mut*^	1.58	0.91
*osk SRS 5*^*mut*^	1.97	1.57
*osk SRS 2*,*3*^*mut*^	1.37	0.81

RNA levels were measured by RT-qPCR.

^a^All transgene mRNA levels are relative to the *osk*^+^ control.

RNA distributions were detected by *in situ* hybridization using two types of probes: tiled short DNA oligonucleotides (smFISH) (Figs 1B, 2B, 3D, 6A, 7A, 7B and 7D) or RNAs synthesized by *in vitro* transcription (Fig 1E and S3 Fig). For smFISH the tiled oligonucleotides were 3’-end labeled with Quasar 670 fluorophore (LGC Biosearch Technologies) and used at 1.5 nM. Assays were performed as described [41]. Immunodetection of OSK protein in fixed ovaries (S4 Fig) was as described [42]. Samples were imaged with a Nikon C2+ laser scanning confocal microscope. For all imaging experiments the samples were obtained from at least five flies, typically many more. Imaging experiments to detect mRNAs by *in situ* hybridization were performed in groups, with all samples from a single comparison set imaged in the same session with identical confocal settings. The sole exception is in Fig 6A, where the panel showing the distribution of *osk* mRNA in a *stau* mutant was obtained from a separate experiment; this image was not used for quantitation. Quantification of *in situ* hybridization data was done with Adobe Photoshop, FIJI or Nikon NIS Elements software, using different strategies for different types of experiments. To analyze anterior *osk* mRNA levels in Fig 3D, all images were copied to a single file then merged to one layer, threshold for the green (RNA) channel was set to 12 (sufficient to eliminate all background signal while retaining a high degree of sensitivity), and each late stage 9 egg chamber was scored for presence or absence of any signal above threshold at each of the two anterior/lateral junctions in the oocyte, producing scores of 0, 1 or 2. FIJI was used to quantitate the degree of anterior oocyte retention in Fig 2B. Areas of similar size were traced at each anterior/lateral junction of individual oocytes following the outline of the oocyte and extending about 5% of the length and width of the oocyte, and a single larger area was traced in the center of the oocyte away from the cortex. Average signal intensities were measured for each area, the values for the two anterior regions were averaged, and the value for the central area (background) was subtracted. All other analyses used Nikon NIS Elements.

To quantitate the degree of oocyte enrichment in early-stage egg chambers, regions of interest (ROIs) of nurse cell cytoplasm and oocyte cytoplasm were drawn. Average signal intensities within the ROIs were calculated from total pixel intensities divided by ROI areas. For quantitation of levels of RNA in the posterior region of stage 9 and 10A oocytes (Fig 6C), the Nikon Elements ROI auto-detect function was used to outline the posterior signal, with background (measured in follicle cells where *osk* is not expressed) subtracted. As an estimate of the fraction of RNA in the anterior region of oocytes (Fig 6D), the lateral cortex of each oocyte (not including the anterior boundary that abuts the nurse cells) was traced, yielding data sets as shown in Fig 6B. The sum of the signal in the first 15% and last 15% of each trace (the anterior lateral regions) was divided by the sum of the first and last 15% plus the central 30% (the posterior region) of each trace to give the value shown in Fig 6D.

### RNA-binding assays with EGL protein

For EGL protein expression, the *egl in vitro* transcription/translation template was PCR amplified from a linearized plasmid template that contains the *egl* ORF using Phusion HF polymerase (NEB) according to the manufacturer’s instructions with a final concentration of 3% DMSO to promote denaturation. PCR cycling conditions were: denaturation at 98°C for 10 sec, 34 amplification cycles with annealing at 50°C + 0.5°C/cycle and extension at 72°C for 1 min, and a final cycle of 72°C for 10 min. The primers introduce a T7 promoter and a Kozak consensus sequence at the 5’ end and a stop codon and a poly(A) tail at the 3’ end (Egl F: 5’ CGA TTT GAA TTC TAA TAC GAC TCA CTA TAG GGA ACA GCC ACC ATG GAG TCC ATG GAG TAC GAG ATG GCA 3’; Egl Rev: 5’ TAT ATA GGA TCC TTT TTT TTT TTT TTT TTT TTT TTT TTT TTT TTA TGT GG GAGA CACA CG CTT CGC GGG 3’). The primer concentration in the PCR reaction was 100 nM to limit amplification of PCR products that were not full-length. PCR products were purified using a MinElute column (MinElute Reaction Cleanup Kit, Qiagen) and quantitated using a Nanodrop.

^35^S-methionine-labeled proteins were produced in rabbit reticulocyte lysates using a T7 quick-coupled transcription/translation (TNT) system (Promega) according to the manufacturer’s instructions, using 400–500 ng of purified PCR product per 50 μl reaction. To estimate protein yield, an aliquot of synthesized protein was passed over a Zeba Spin desalting column (Thermo Fisher Scientific) to remove unincorporated methionine and counted in a scintillation counter against a serial dilution of ^35^S- methionine. Protein yield was found to be in the femtomolar range. Proteins were analyzed in 4–20% Mini-PROTEAN TXG gels (Bio-Rad), dried, exposed to a PhosphorImager screen (Fuji), scanned in a Typhoon Imager (GE) and analyzed using Quantity One software (Bio-Rad).

To prepare templates for transcription of RNA substrates, sequences were PCR amplified with primers that append a 5’ T7 promoter and cloned into the *Eco*RI and *Bam*HI sites of the pUC-19 vector, to which the S1mx4 aptamer [23] was added using the *Pst*I and *Hin*dIII sites, respectively. The S1mx4 aptamer by itself was cloned into pBluescript KS + (5’ *Pst*I, 3’ *Hin*dIII). RNAs, with 4 copies of the S1mx4 aptamer at the 3’, or the S1mx4 aptamer by itself, were generated by *in vitro* transcription (Megascript T7 kit, Invitrogen) from *Hin*dIII linearized templates, and purified using Zymo RNA Clean and Concentrator columns (ZYMO Research) according to instructions and concentration determined by measuring using the Nanodrop.

Before immobilizing RNAs on magnetic streptavidin beads, they were incubated in 1x DXB buffer (30 mM Hepes at pH 7.3, 50 mM KCl, 2.5 mM MgCl_2_, 250 mM sucrose and 1 mM DTT, 2 μg of RNA in a 20 μl reaction) at 56°C for 5 min, followed by 10 min at 37°C and 15 min at 20°C to allow refolding [23]. The refolded RNA was bound to 50 μl of washed magnetic Dynabeads (Thermo Fisher Scientific) in 100 μl of 1x DXB buffer and 0.01% NP-40 (DXB+NP-40) and RNAse inhibitor (SUPERaseIn, Thermo Fisher Scientific) in DNA LoBind tubes (Eppendorf) in a Thermomixer (Eppendorf) at 4°C for 2 hours at 1000 rpm. For a given set of experiments, the magnetic beads were washed in one batch (3 washes with Buffer A: DEPC treated 0.1M NaOH and 0.05 M NaCl, 3 washes with DEPC treated 0.1 M NaCl, and 3 washes with DXB+NP-40) and distributed into individual tubes before the addition of the different RNAs.

After binding, the unbound RNAs were removed, and streptavidin-bound RNAs were rinsed twice with DXB+NP-40 on the magnetic rack, after which *in vitro* transcribed/translated EGL protein (10–12.5 μl of a 50 μl *in vitro* transcription/translation reaction per RNA) was added in 100 μl DXB containing 1x EDTA-free protease inhibitor (Roche) and RNAse inhibitor and incubated on a Thermomixer for 90 min at 1000 rpm. Unbound RNAs were run on an agarose gel to ascertain even input.

After binding of the protein to the affinity-bound RNAs, beads were washed 6 times with DXB+NP-40 buffer on the rack, after which the bead- bound protein was boiled off by adding 100 μl of 1x Laemmli buffer and incubating for 15 minutes on a Thermomixer at 100°C and 1000 rpm. Tubes were spun briefly before loading sample on a protein gel to remove particulate matter. An aliquot of the EGL protein used for the binding experiment was run as an input control.

To establish the level of background binding an aptamer-only control was used. This reveals the level of EGL bound to both aptamer and beads. Although not included in Fig 3 we also tested, in separate experiments, EGL binding to beads alone (a no RNA control). Typically, observed binding of EGL to the beads in the absence of RNA was less than 10% of the level of binding to beads plus aptamer.

### RNA structure predictions

Predictions of RNA folding were performed in the Mfold Web Server (http://unafold.rna.albany.edu/?q=mfold/RNA-Folding-Form) [43].

### Reagents for RNA co-immunoprecipitation (RIP) assays

Four plasmids were used. The first expressed an N-terminal 3xFLAG-tagged STAU protein (3xFLAG-STAU). The second expressed Firefly *luciferase* reporter mRNA with the wild-type and mutant versions of the *osk* 3’ UTR described above (Firefly *luciferase-osk 3’ UTR*). The third expressed *Renilla luciferase* and served as a transfection control (*Renilla luciferase*). The fourth plasmid was pSP72, a transfection carrier DNA.

Luciferase reporters were cloned into pRmHa3 [44] and, thus, were under the control of the metal-inducible metallothionein promoter. The wild-type and mutant *osk 3’UTR* DNA constructs were inserted into the Firefly *luciferase* reporter vector at the *Bam*HI and *Sal*I restriction sites. All *osk 3′ UTR* constructs were truncated at the 3’ end, with the final 68 nt of the *osk* mRNA removed. 3xFLAG-STAU was derived from pAc5.1/V5-His (Thermo Fisher Scientific), which carries the Actin5C promoter. To produce 3xFLAG-STAU, wild-type *stau* isoform C was amplified by PCR from whole embryo 0–3 hours extract and inserted into the vector at the *Eco*RV and *Xba*I restriction sites.

*Drosophila* S2 tissue culture cells were maintained at 25°C in Express Five SFM (Fisher Scientific) containing 100 units/mL penicillin, 100 μg/mL streptomycin and 16mM glutamine. A mixture of 250 ng Firefly *luciferase-osk 3′ UTR* plasmid, 50 ng *Renilla luciferase* plasmid, 250 ng 3xFLAG-STAU plasmid was transfected into 4 mL of S2 cells at a density of 1.5 x 10^6^ cells/mL using 6 μL X-tremeGENE 9 DNA Transfection Reagent (Roche), according to the manufacturer’s instructions. The expression of luciferase reporters was induced 3 hours post transfection through the addition of copper sulfate to a final concentration of 0.5 mM.

72 hours post-transfection S2 cells—resulting in a total of 6 x 10^6^ cells/mL—were harvested, incubated in lysis buffer (150 mM KCl, 20 mM Hepes pH 7.4, 1 mM MgCl_2_, protease inhibitor, DTT, and 0.3% Triton X-100) for 25 minutes on ice (4°C), then centrifuged for 15 minutes 13000 rpm 4°C, and supernatant was recovered. The protein concentration of the lysate was determined with a Bio-Rad Protein Assay Dye Reagent. The lysate was diluted to 8.5 μg/μL with lysis buffer.

### RNA co-immunoprecipitation (RIP)

Each IP used 5 μL of anti-FLAG M2 beads, which were washed four times with lysis buffer (150 mM KCl, 20 mM Hepes pH 7.4, 1 mM MgCl_2_, protease inhibitor, DTT, and 0.1% Triton X-100). 90 μL of diluted, cleared supernatant was added to each tube of beads and incubated for 3 hours at 4°C with end-over-end rotation. Beads were washed five times with lysis buffer (150 mM KCl, 20 mM Hepes pH 7.4, 1 mM MgCl_2_, 0.1% Triton X-100, protease inhibitors, DTT). 50 μL of lysis buffer was added to the beads. The bead volume was then split in half: (1) for RNA isolation with TRIzol and (2) for protein quantification. In addition, 50 μL of extract was saved to measure RNA ‘input’ and 10 μL for ‘input’ protein.

A Western blot was performed on the lysate extract of the input and RIP to quantify the efficiency of IP of 3xFLAG-STAU by anti-FLAG M2 beads. 10 to 15 μL of lysate from RIP was resolved via SDS-PAGE. The proteins were transferred to PVDF membrane, which was blocked at room temperature for 1 hour with 2% milk in PBST (1x PBS + 0.1% Tween20). The blots were incubated overnight with anti-FLAG M2 antibodies (Sigma-Aldrich) at 4°C and then incubated with HRP-conjugated secondary antibody (1:5000) at room temperature for 1 to 3 hours. The blots were developed with ECL Plus detection reagents (MilliporeSigma Luminata Crescendo) and then imaged and quantified using the VersaDoc Imaging System (Bio-Rad). The relative levels of 3xFLAG-STAU from RIP were determined using a standard curve.

RNA was isolated from RNA co-immunoprecipitation by adding 9:1 of TRIzol reagent (Invitrogen) to bead volume and the RNA was purified according to manufacturer’s instructions. The isolated RNA was quantified using a NanoDrop Fluorospectrometer.

The RNA was treated with DNAse I, according to Invitrogen’s instructions with the following minor modifications. 1 μL of 25 mM EDTA was substituted with DEPC-treated water, 2.5 μg of RNA ‘input’ and 8 μL of RNA from IP were used instead of 1 μg of RNA. In addition to the manufacturer’s protocol, the DNAse-treated RNA was heated at 80°C for 4 minutes. The RNA was used to generate cDNA through reverse transcription with SuperScript IV reverse transcriptase (Invitrogen) and random hexamers (Thermo Fisher Scientific) by following the manufacturer’s instructions. The cDNA was subjected to quantitative real-time PCR using the SensiFast SYBR PCR mix (Bioline) and primers against the Firefly and *Renilla* luciferase ORFs.

Relative levels of the Firefly and *Renilla* transcripts were determined using a standard curve. The STAU-binding enrichment was calculated by first normalizing Firefly *luciferase-osk 3′UTR* expression to *Renilla luciferase* expression from IP, then by normalizing to input, and finally by normalizing to Firefly *luciferase*-only vector.

### Numerical data

Numerical data underlying all graphs in the figures is provided in S1 File.

### Statistical methods

Reproducibility was confirmed by performing independent experiments. Biochemical experiments were repeated a minimum of three times. Imaging experiments, which were performed at least twice, involved examination of multiple individual egg chambers in each experiment. Here, repetition served to reveal any technical problems, and the large number of individual egg chambers scored in each experiment ensured consistency and reproducibility. Egg laying experiments were performed at least three times. Rates of egg laying often show circadian variation, but because each experiment consisted of egg collections over several days, this source of variation was minimized. The experiments were not randomized, and no statistical method was used to predetermine sample size. One-way ANOVA was used to ask if there were significant differences among data sets with three or more variables. For *post hoc* analyses, Shapiro-Wilk normality tests were first performed. If the test failed to reject normality, Student’s *t* tests were used for *post hoc* analysis. If normality was rejected, Wilcoxon rank sum tests were used for *post hoc* analysis. The particular tests used are indicated in the figure legends.

## Supporting information

S1 Fig Phylogenetic comparison of the SL2a transport and anchoring signal (TAS). Diagrams of a central portion of the SL2a region of the *osk* mRNA 3’ UTR from multiple *Drosophila* species (certain species closely related to *D*. *melanogaster* and typically identical in SL2a signal sequence are not included). The highly conserved transport and anchoring signal region is in black for all, and the less well conserved flanking regions from non-*melanogaster* species are in grey. The consensus signal, supported by mutational analysis [11] is at left, with highly variable positions (those found in multiple species) in blue. Within the individual structures, bases at the variable positions are in blue, and bases at positions that differ less frequently are outlined in blue.
(PDF)

S2 Fig Effects of SRS mutations on predicted folding of SL2. (A) Predicted free energies of folding for SL2, either wild type or with SRS mutations. Values for all predicted folds within 10% of the lowest ΔG are listed. The presence of the correctly folded TAS for each predicted fold is indicated. B. Comparison of examples of folding options for SRS 3 mutants with the wild-type fold. Sequences that comprise the SL2a TAS when correctly folded are highlighted in green.
(PDF)

S3 Fig Axial patterning of embryos from *osk* RNA null mothers with genomic *osk* transgenes as indicated. Wild-type *osk* activity results in embryos with the normal 8 abdominal segments. Fewer abdominal segments are indicative of decreasing levels of *osk* activity. N values were greater than 300 for all but the SRS 3 mutant (n = 45) which, compared to wild type, produced fewer eggs that frequently failed to develop.
(PDF)

S4 Fig OSK protein expression at the posterior pole of SRS mutants. Total signal intensities were measured for OSK protein at the posterior pole of stage 9/10A egg chambers with the results presented in dot plot format. Statistical significance was evaluated by one-way ANOVA with F (8,247) = 94.21, P < 2 x 10^−70^. Normality was rejected (Bonferroni corrected Shapiro-Wilk test), therefore the Wilcoxon rank sum test was used for *post hoc* analysis. ***: P < 0.01, *: P < 0.1, NS: not significant. N values for all genotypes were 19 or greater.
(PDF)

S5 Fig Transport to the oocyte is not disrupted for *osk* mRNA with mutations in SRSs 1 and 5. *osk* mRNAs, either a control (*osk*^+^) or with mutations in SRSs 1 and 5 (*SRS 1*,*5*^*mut*^ and *SRS 1*,*5*^*comp*^) were detected by *in situ* hybridization. The RNA levels in nurse cells and oocytes were quantitated to obtain average per unit area levels and plotted as ratios of oocyte average/nurse cell average in dot plot format. Statistical significance was evaluated by one-way ANOVA with F (2,87) = 2.32, P = 0.105. Thus, the null hypothesis of no significant difference between the means was accepted. N values for all genotypes were 30.
(PDF)

S1 File Numerical data for figures. An Excel file with underlying data for figures, arranged by figure and panel.
(XLSX)

## References

[1] Lipshitz HD, Smibert CA. Mechanisms of RNA localization and translational regulation. Curr Opin Genet Dev. 2000; 10:476–488. 10.1016/s0959-437x(00)00116-7 10980424

[2] St Johnston D. Moving messages: the intracellular localization of mRNAs. Nat Rev Mol Cell Biol. 2005; 6:363–375. 10.1038/nrm1643 15852043

[3] Lehmann R. Germ Plasm Biogenesis-An Oskar-Centric Perspective. Curr Top Dev Biol. 2016; 116:679–707. 10.1016/bs.ctdb.2015.11.024 26970648 PMC4959550

[4] Ephrussi A, Lehmann R. Induction of germ cell formation by *oskar*. Nature. 1992; 358:387–392. 10.1038/358387a0 1641021

[5] Smith JL, Wilson JE, Macdonald PM. Overexpression of *oskar* Directs Ectopic Activation of *nanos* and Presumptive Pole Cell Formation in Drosophila Embryos. Cell. 1992; 70:849–859. 10.1016/0092-8674(92)90318-7 1516136

[6] Jenny A, Hachet O, Závorszky P et al. A translation-independent role of *oskar* RNA in early *Drosophila* oogenesis. Development. 2006; 133:2827–2833. 10.1242/dev.02456 16835436

[7] Kanke M, Jambor H, Reich J et al. *oskar* RNA plays multiple noncoding roles to support oogenesis and maintain integrity of the germline/soma distinction. RNA. 2015; 21:1096–1109. 10.1261/rna.048298.114 25862242 PMC4436663

[8] Kim-Ha J, Webster PJ, Smith JL, Macdonald PM. Multiple RNA regulatory elements mediate distinct steps in localization of *oskar* mRNA. Development. 1993; 119:169–178. 8275853 10.1242/dev.119.1.169

[9] Kim J, Lee J, Lee S, Lee B, Kim-Ha J. Phylogenetic comparison of *oskar* mRNA localization signals. Biochem Biophys Res Commun. 2014; 444:98–103. 10.1016/j.bbrc.2014.01.021 24440702

[10] Jambor H, Mueller S, Bullock SL, Ephrussi A. A stem-loop structure directs *oskar* mRNA to microtubule minus ends. RNA. 2014; 20:429–439. 10.1261/rna.041566.113 24572808 PMC3964905

[11] Ryu YH, Kenny A, Gim Y, Snee M, Macdonald PM. Multiple *cis*-acting signals, some weak by necessity, collectively direct robust transport of *oskar* mRNA to the oocyte. J Cell Sci. 2017; 130:3060–3071. 10.1242/jcs.202069 28760927 PMC5612174

[12] Serano TL, Cohen RS. A small predicted stem-loop structure mediates oocyte localization of *Drosophila K10* mRNA. Development. 1995; 121:3809–3818. 8582290 10.1242/dev.121.11.3809

[13] Bullock SL, Ish-Horowicz D. Conserved signals and machinery for RNA transport in *Drosophila* oogenesis and embryogenesis. Nature. 2001; 414:611–616. 10.1038/414611a 11740552

[14] Dienstbier M, Boehl F, Li X, Bullock SL. Egalitarian is a selective RNA-binding protein linking mRNA localization signals to the dynein motor. Genes Dev. 2009; 23:1546–1558. 10.1101/gad.531009 19515976 PMC2704466

[15] Sanghavi P, Laxani S, Li X, Bullock SL, Gonsalvez GB. Dynein Associates with *oskar* mRNPs and Is Required For Their Efficient Net Plus-End Localization in *Drosophila* Oocytes. PLoS One. 2013;8:e80605. 10.1371/journal.pone.0080605 24244700 PMC3823658

[16] Kim-Ha J, Smith JL, Macdonald PM. *oskar* mRNA Is Localized to the Posterior Pole of the Drosophila Oocyte. Cell. 1991; 66:23–35. 10.1016/0092-8674(91)90136-m 2070416

[17] Ephrussi A, Dickinson LK, Lehmann R. *oskar* Organizes the Germ Plasm and Directs Localization of the Posterior Determinant *nanos*. Cell. 1991; 66:37–50. 10.1016/0092-8674(91)90137-n 2070417

[18] Micklem DR, Adams J, Grunert S, St Johnston D. Distinct roles of two conserved Staufen domains in *oskar* mRNA localization and translation. EMBO J. 2000; 19:1366–1377. 10.1093/emboj/19.6.1366 10716936 PMC305677

[19] Laver JD, Li X, Ancevicius K et al. Genome-wide analysis of Staufen-associated mRNAs identifies secondary structures that confer target specificity. Nucleic Acids Res. 2013; 41:9438–9460. 10.1093/nar/gkt702 23945942 PMC3814352

[20] Cheong C, Varani G, Tinoco I Jr. Solution structure of an unusually stable RNA hairpin, 5’GGAC(UUCG)GUCC. Nature. 1990; 346:680–682. 10.1038/346680a0 1696688

[21] Uhlenbeck OC. Tetraloops and RNA folding. Nature. 1990; 346:613–614. 10.1038/346613a0 1696683

[22] Srisawat C, Engelke DR. Streptavidin aptamers: Affinity tags for the study of RNAs and ribonucleoproteins. RNA. 2001; 7:632–641. 10.1017/s135583820100245x 11345441 PMC1370116

[23] Leppek K, Stoecklin G. An optimized streptavidin-binding RNA aptamer for purification of ribonucleoprotein complexes identifies novel ARE-binding proteins. Nucleic Acids Res. 2014;42:e13. 10.1093/nar/gkt956 24157833 PMC3902943

[24] Liu Y, Salter HK, Holding AN et al. Bicaudal-D uses a parallel, homodimeric coiled coil with heterotypic registry to coordinate recruitment of cargos to dynein. Genes Dev. 2013; 27:1233–1246. 10.1101/gad.212381.112 23723415 PMC3690397

[25] St Johnston D, Beuchle D, Nüsslein-Volhard C. *staufen*, a Gene Required to Localize Maternal RNAs in the Drosophila Egg. Cell. 1991; 66:51–63. 10.1016/0092-8674(91)90138-o 1712672

[26] Sanghavi P, Liu G, Veeranan-Karmegam R, Navarro C, Gonsalvez GB. Multiple Roles for Egalitarian in Polarization of the *Drosophila* Egg Chamber. Genetics. 2016; 203:415–432. 10.1534/genetics.115.184622 27017624 PMC4858789

[27] Hoogenraad CC, Akhmanova A, Howell SA et al. Mammalian Golgi-associated Bicaudal-D2 functions in the dynein-dynactin pathway by interacting with these complexes. EMBO J. 2001; 20:4041–4054. 10.1093/emboj/20.15.4041 11483508 PMC149157

[28] Hoogenraad CC, Wulf P, Schiefermeier N et al. Bicaudal D induces selective dynein-mediated microtubule minus end-directed transport. EMBO J. 2003; 22:6004–6015. 10.1093/emboj/cdg592 14609947 PMC275447

[29] Splinter D, Razafsky DS, Schlager MA et al. BICD2, dynactin, and LIS1 cooperate in regulating dynein recruitment to cellular structures. Mol Biol Cell. 2012; 23:4226–4241. 10.1091/mbc.E12-03-0210 22956769 PMC3484101

[30] Bullock SL, Ringel I, Ish-Horowicz D, Lukavsky PJ. A’-form RNA helices are required for cytoplasmic mRNA transport in *Drosophila*. Nat Struct Mol Biol. 2010;17:703–709. 10.1038/nsmb.1813 20473315 PMC2997434

[31] Yadav DK, Zigáčková D, Zlobina M et al. Staufen1 reads out structure and sequence features in ARF1 dsRNA for target recognition. Nucleic Acids Res. 2020; 48:2091–2106. 10.1093/nar/gkz1163 31875226 PMC7038937

[32] Roegiers F, Jan YN. Staufen: a common component of mRNA transport in oocytes and neurons. Trends Cell Biol. 2000; 10:220–224. 10.1016/s0962-8924(00)01767-0 10802537

[33] Park E, Maquat LE. Staufen-mediated mRNA decay. Wiley Interdiscip Rev RNA. 2013; 4:423–435. 10.1002/wrna.1168 23681777 PMC3711692

[34] Ren Z, Veksler-Lublinsky I, Morrissey D, Ambros V. Staufen Negatively Modulates MicroRNA Activity in *Caenorhabditis elegans*. G3 (Bethesda). 2016; 6:1227–1237. 10.1534/g3.116.027300 26921297 PMC4856075

[35] Dugré-Brisson S, Elvira G, Boulay K, Chatel-Chaix L, Mouland AJ, DesGroseillers L. Interaction of Staufen1 with the 5’ end of mRNA facilitates translation of these RNAs. Nucleic Acids Res. 2005; 33:4797–4812. 10.1093/nar/gki794 16126845 PMC1193567

[36] Reveal B, Yan N, Snee MJ, Pai C-I, Gim Y, Macdonald PM. BREs Mediate Both Repression and Activation of *oskar* mRNA Translation and Act In *trans*. Dev Cell. 2010; 18:496–502. 10.1016/j.devcel.2009.12.021 20230756 PMC2841435

[37] Larkin A, Marygold SJ, Antonazzo G et al. FlyBase: updates to the Drosophila melanogaster knowledge base. Nucleic Acids Res. 2021;49: D899–D907. 10.1093/nar/gkaa1026 33219682 PMC7779046

[38] Kim G, Pai C-I, Sato K, Person MD, Nakamura A, Macdonald PM. Region-Specific Activation of *oskar* mRNA Translation by Inhibition of Bruno-Mediated Repression. PLoS Genet. 2015;11:e1004992. 10.1371/journal.pgen.1004992 25723530 PMC4344327

[39] Martin SG, St Johnston D. A role for *Drosophila* LKB1 in anterior-posterior axis formation and epithelial polarity. Nature. 2003; 421:379–384. 10.1038/nature01296 12540903

[40] Schmittgen TD, Livak KJ. Analyzing real-time PCR data by the comparative C(T) method. Nat Protoc. 2008; 3:1101–1108. 10.1038/nprot.2008.73 18546601

[41] Abbaszadeh EK, Gavis ER. Fixed and live visualization of RNAs in *Drosophila* oocytes and embryos. Methods. 2016; 98:34–41. 10.1016/j.ymeth.2016.01.018 26827935 PMC4808400

[42] Ryu YH, Macdonald PM. RNA sequences required for the noncoding function of *oskar* RNA also mediate regulation of Oskar protein expression by Bicoid Stability Factor. Dev Biol. 2015; 407:211–223. 10.1016/j.ydbio.2015.09.014 26433064 PMC4663177

[43] Zuker M. Mfold web server for nucleic acid folding and hybridization prediction. Nucleic Acids Res. 2003; 31:3406–3415. 10.1093/nar/gkg595 12824337 PMC169194

[44] Mohan RD, Dialynas G, Weake VM et al. Loss of Drosophila Ataxin-7, a SAGA subunit, reduces H2B ubiquitination and leads to neural and retinal degeneration. Genes Dev. 2014; 28:259–272. 10.1101/gad.225151.113 24493646 PMC3923968

