# Supplementary figures and images for: Opposing roles for Egalitarian and Staufen in transport, anchoring and localization of oskar mRNA in the Drosophila oocyte
Source: PLoS Genet. 2021 Apr 2;17(4):e1009500. doi: 10.1371/journal.pgen.1009500 (PMC8046350; doi:10.1371/journal.pgen.1009500)

**N** Variable  
**A** Differs from consensus

U-A  
 A-U  
 U-A  
 G-C  
 U-A  
 C-G  
 U  
 C-G  
 U-A  
 C-G  
 G-C  
 U-A  
 A-U  
 C-G

Consensus

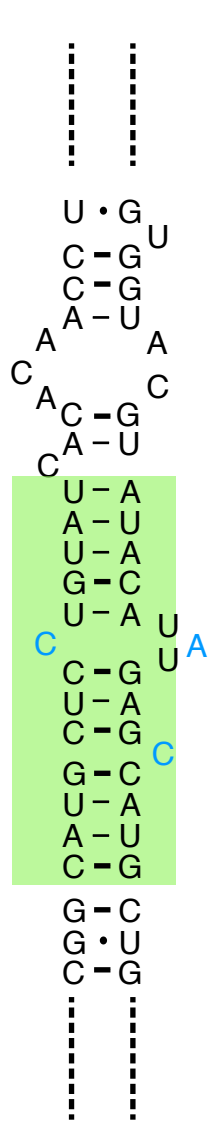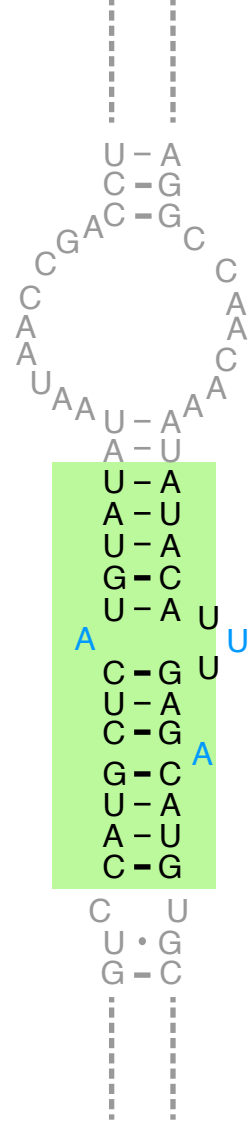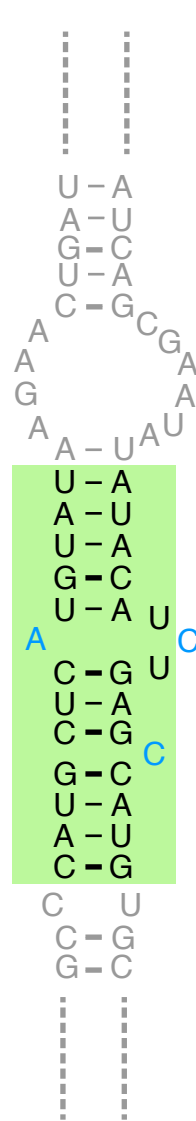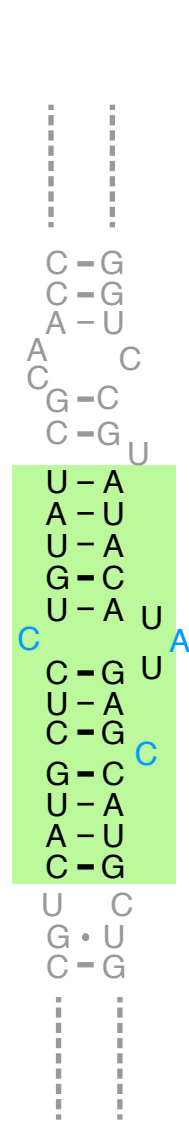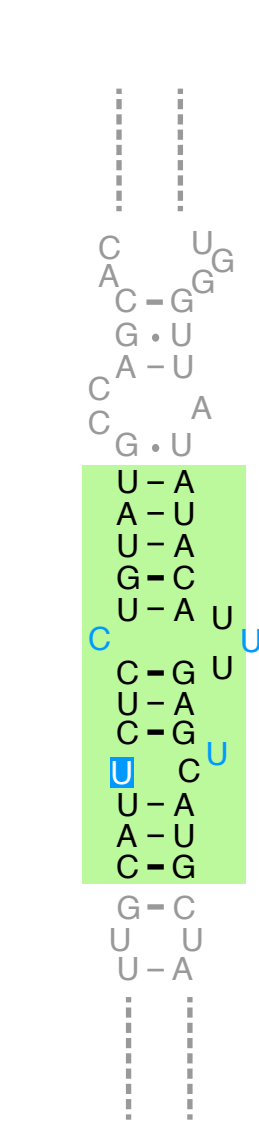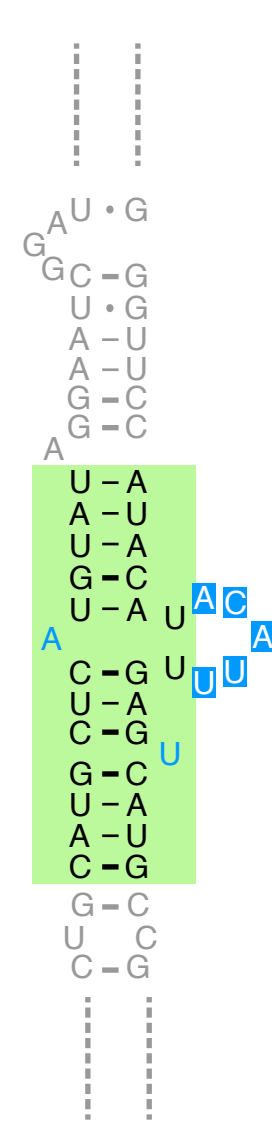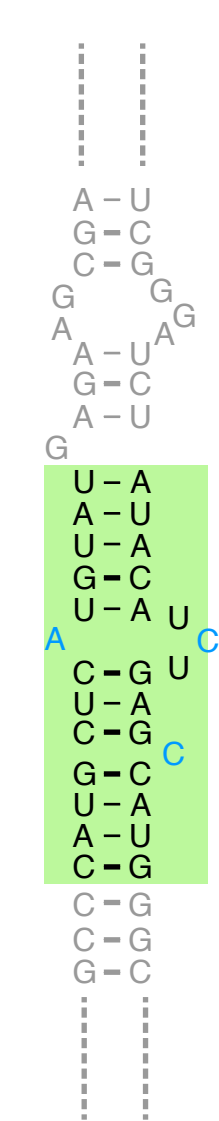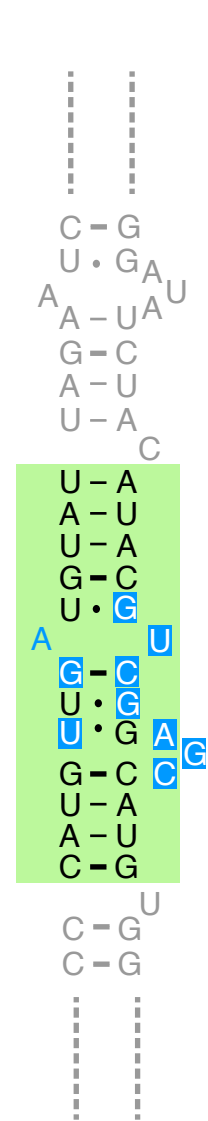

Supplement: S1 Fig — Diagrams of a central portion of the SL2a region of the osk mRNA 3’ UTR from multiple Drosophila species (certain species closely related to D. melanogaster and typically identical in SL2a signal sequence are not included). The highly conserved transport and anchoring signal region is in black for all, and the less well conserved flanking regions from non-melanogaster species are in grey. The consensus signal, supported by mutational analysis [11] is at left, with highly variable positions (those found in multiple species) in blue. Within the individual structures, bases at the variable positions are in blue, and bases at positions that differ less frequently are outlined in blue. (PDF) [file pgen.1009500.s001.pdf]

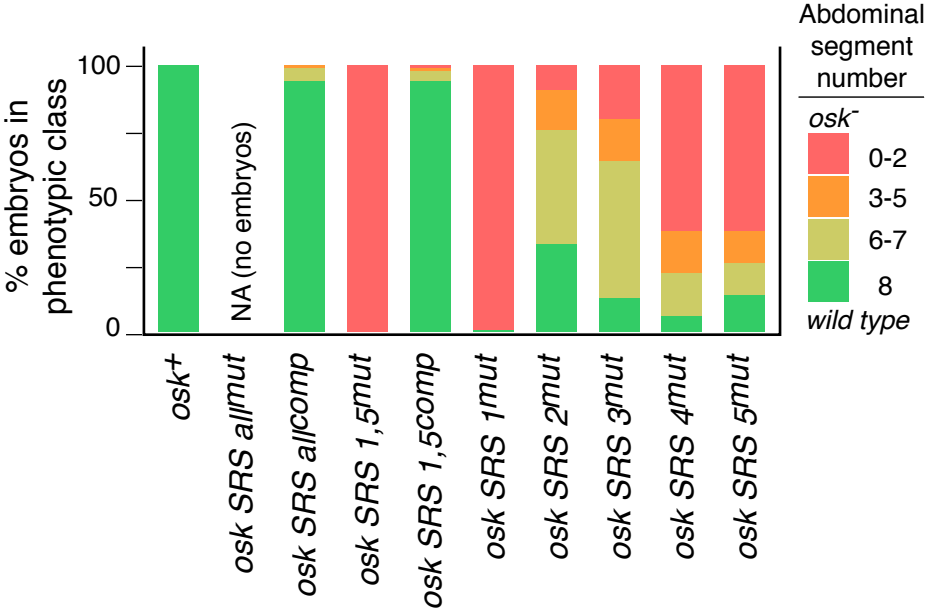

Supplement: S3 Fig — Wild-type osk activity results in embryos with the normal 8 abdominal segments. Fewer abdominal segments are indicative of decreasing levels of osk activity. N values were greater than 300 for all but the SRS 3 mutant (n = 45) which, compared to wild type, produced fewer eggs that frequently failed to develop. (PDF) [file pgen.1009500.s003.pdf]

OskHA signal at  
oocyte posterior  
(arbitrary units)

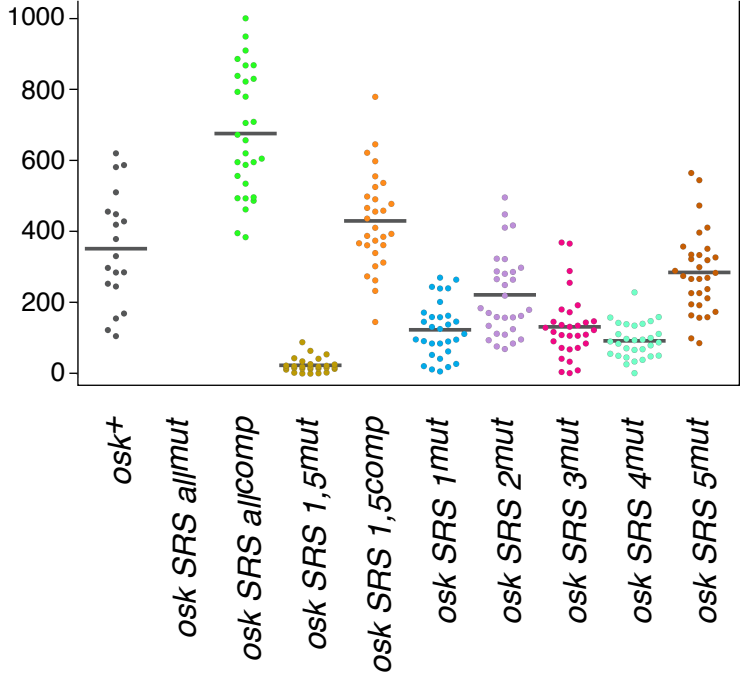

Supplement: S4 Fig — Total signal intensities were measured for OSK protein at the posterior pole of stage 9/10A egg chambers with the results presented in dot plot format. Statistical significance was evaluated by one-way ANOVA with F (8,247) = 94.21, P < 2 x 10^−70^. Normality was rejected (Bonferroni corrected Shapiro-Wilk test), therefore the Wilcoxon rank sum test was used for post hoc analysis. ***: P < 0.01, *: P < 0.1, NS: not significant. N values for all genotypes were 19 or greater. (PDF) [file pgen.1009500.s004.pdf]

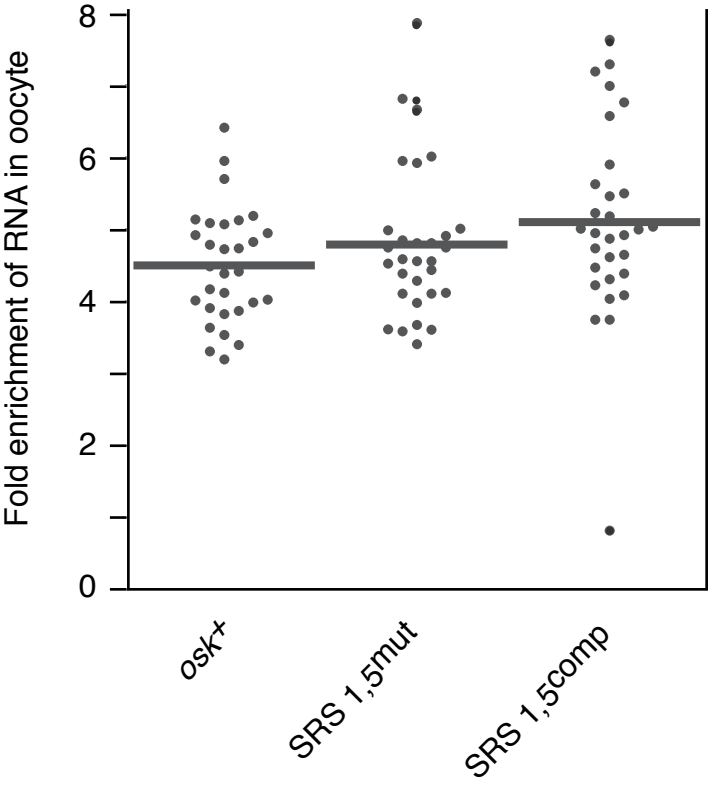

Supplement: S5 Fig — osk mRNAs, either a control (osk+) or with mutations in SRSs 1 and 5 (SRS 1,5mut and SRS 1,5comp) were detected by in situ hybridization. The RNA levels in nurse cells and oocytes were quantitated to obtain average per unit area levels and plotted as ratios of oocyte average/nurse cell average in dot plot format. Statistical significance was evaluated by one-way ANOVA with F (2,87) = 2.32, P = 0.105. Thus, the null hypothesis of no significant difference between the means was accepted. N values for all genotypes were 30. (PDF) [file pgen.1009500.s005.pdf]
