# Supplementary material for: Opposing roles for Egalitarian and Staufen in transport, anchoring and localization of oskar mRNA in the Drosophila oocyte
Source: PLoS Genet. 2021 Apr 2;17(4):e1009500. doi: 10.1371/journal.pgen.1009500 (PMC8046350; doi:10.1371/journal.pgen.1009500)

A

Properties of predicted SL2 folds (within 10% of most stable)

	wt ΔG	wt TAS fold?	SRSs 2,3,4 mutated ΔG	SRSs 2,3,4 mutated TAS fold?	SRSs 2,3 mutated ΔG	SRSs 2,3 mutated TAS fold?	SRS 2 mutated ΔG	SRS 2 mutated TAS fold?	SRS 3 mutated ΔG	SRS 3 mutated TAS fold?	SRS 4 mutated ΔG	SRS 4 mutated TAS fold?
SRS mutant	-86.20	yes	-60.27	no	-67.30	no	-75.50	yes	-74.90	no	-76.90	yes
			-59.51	no	-66.03	no	-72.40	no	-71.40	no	-72.84	yes
			-59.11	no	-65.90	no	-71.18	no	-70.45	yes	-71.14	yes
			-58.62	no	-65.73	no	-70.15	no	-70.02	no	-70.70	yes
			-56.07	no	-64.60	no	-69.90	no	-68.10	no	-70.74	yes
			-54.62	no	-63.27	no	-68.23	no				
			-54.32	no	-62.00	no						
					-61.53	no						
					-61.09	no						
SRS mutant + compensatory changes			-84.50	yes	-85.30	yes	-86.60	yes	-84.90	yes	-85.40	yes
							-80.70	yes				

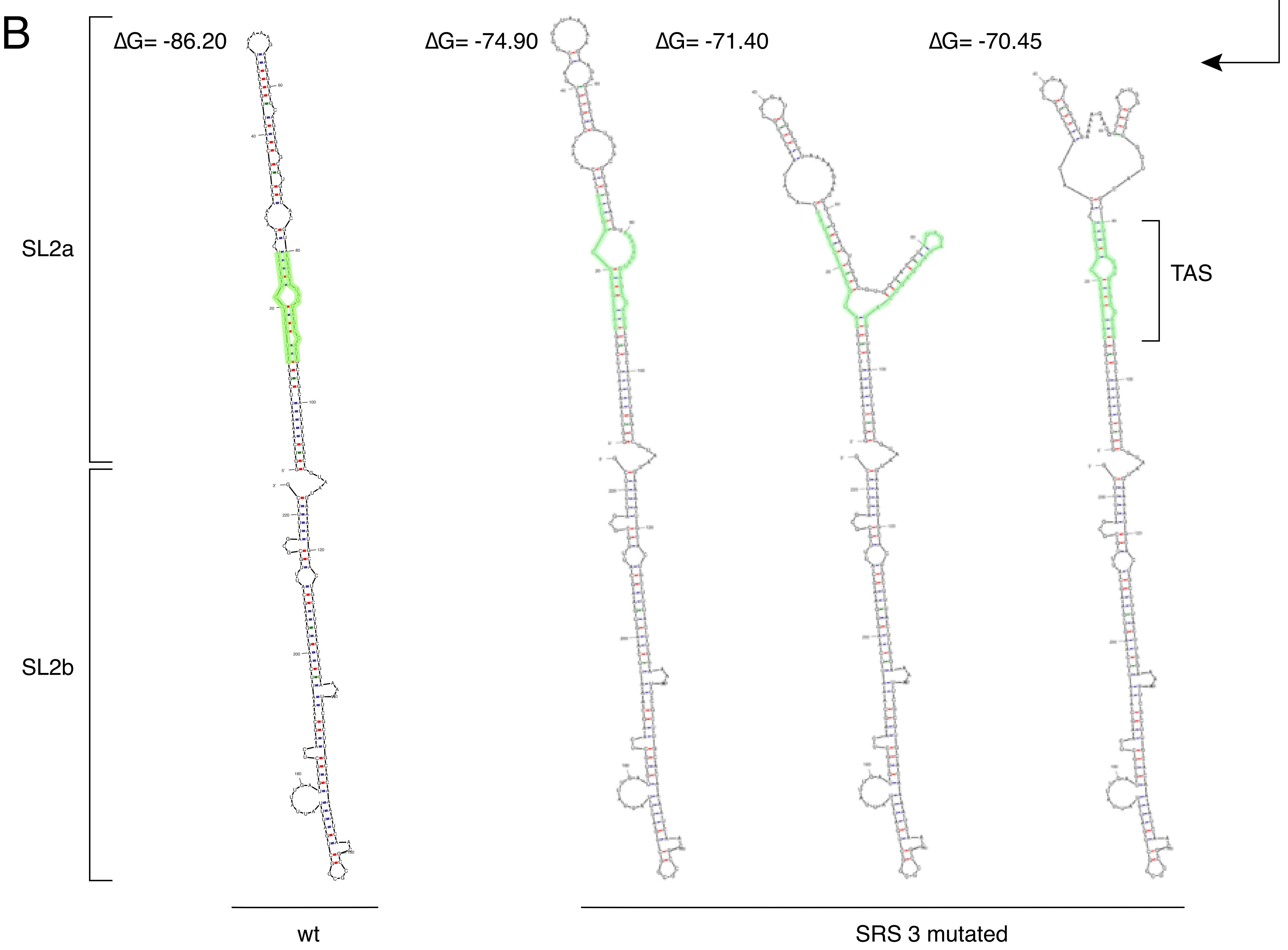

Supplement: S2 Fig — (A) Predicted free energies of folding for SL2, either wild type or with SRS mutations. Values for all predicted folds within 10% of the lowest ΔG are listed. The presence of the correctly folded TAS for each predicted fold is indicated. B. Comparison of examples of folding options for SRS 3 mutants with the wild-type fold. Sequences that comprise the SL2a TAS when correctly folded are highlighted in green. (PDF) [file pgen.1009500.s002.pdf]
